# Maternal high-salt intake induces sex-specific liver injury in offspring: integrative transcriptomic and therapeutic investigation

**DOI:** 10.3389/fnut.2025.1657934

**Published:** 2025-09-16

**Authors:** Xiuli Chen, Rui Chen, Wanyu Song, Li Wang, Haiying Wu

**Affiliations:** Department of Gynecology and Obstetrics, Henan Provincial People's Hospital, People's Hospital of Zhengzhou University, School of Clinical Medicine of Henan University, Zhengzhou, China

**Keywords:** maternal nutrition, high-salt diet, liver injury, transcriptomics, sexual dimorphism

## Abstract

**Introduction:**

Maternal diet is a key determinant of fetal organ development, and excessive salt intake has been increasingly linked to long-term metabolic disorders. This study investigated the effects of maternal high-salt diet (HSD) on offspring liver health, with a focus on sex-specific structural and molecular alterations.

**Methods:**

Pregnant C57BL/6J mice were fed either standard chow or an HSD (4% NaCl) during gestation and lactation. At 4 weeks of age, offspring livers were analyzed using histology, RNA sequencing, immunofluorescence, and biochemical assays. Connectivity Map (CMap) was applied to identify candidate therapeutic compounds.

**Results:**

Maternal HSD significantly increased liver weight and hepatocyte size in offspring, with more pronounced effects in males. RNA sequencing identified 466 differentially expressed genes (DEGs) in males and 309 in females, indicating sex-dependent dysregulation of pathways related to metabolism, fibrosis, inflammation, and apoptosis. Masson's trichrome staining revealed increased collagen deposition, particularly in males, consistent with transcriptomic signatures of fibrosis. Immunofluorescence confirmed upregulation of apoptosis-related markers. CMap analysis identified potential therapeutic agents, including ketoprofen and BRD-K88741031, which alleviated HSD-induced hepatic injury in preliminary validation.

**Discussion:**

Maternal high-salt intake induces sex-specific structural and molecular liver injury in offspring, highlighting the developmental origins of liver disease. These findings emphasize the importance of maternal nutrition in shaping offspring hepatic health and suggest potential pharmacological strategies to counteract early-life dietary insults.

## 1 Introduction

Salt, as a traditional seasoning, facilitates essential physiological processes in the body (1). Sodium in table salt plays a crucial role in maintaining nutrient absorption, muscle movement, nerve transmission, cardiopulmonary function, and metabolism (2, 3). In recent years, a high-salt diet (HSD) has emerged as a significant global public health issue, contributing to 3 million deaths and 70 million illnesses worldwide in 2017 alone (4). The World Health Organization (WHO) recommends that adults consume less than 5 g of iodized salt daily. Studies indicate that salt consumption in most countries averages 9–12 g/day, exceeding the recommended daily limit of 5 g/day (5). Statistics indicate that condiments contribute to 80% of our residents' sodium intake, with excessive intake resulting from the frequent use of diverse condiments (6). Excessive sodium intake can lead to various health issues, including kidney disease (7), cardiovascular disease (8, 9), chronic inflammation (10, 11), autoimmune disease (12, 13), cognitive disorder (14, 15), and cancer (16). Therefore, excessive sodium intake is a prevalent issue that poses a significant threat to human health.

Adverse factors during pregnancy and lactation, such as maternal malnutrition, overnutrition, and illness, can impact fetal development (17–19). There is mounting evidence that adult hypertension may originate early in life, and adverse events during early development may pre-dispose individuals to the increased risk of cardiovascular and other adult diseases in offspring (20, 21). Some pregnant women tend to select foods with higher salt content due to taste changes (22, 23). Recently, there has been increasing attention on the impacts of maternal high-salt intake on offspring, including cardiovascular function and liver (24–26). Elevated salt intake in pregnant women tends to influence uterine arterial reactivity, perfusion pressure, and potentially impacts offspring cardiovascular system development (27, 28). Moreover, maternal exposure to high salt concentrations in the environment, in rats, and in chicken embryos has demonstrated evident signs of liver fibrosis and increased mortality risk in liver cells (25). However, the mechanisms underlying how an HSD during fetal and infant stages damages liver tissue and contributes to liver disease remain poorly understood.

Sex differences in hepatic physiology and pathophysiology are well-established, with males and females exhibiting distinct susceptibilities to metabolic stress, inflammation, and fibrosis (29–31). For example, males are more susceptible to liver fibrosis and steatosis, whereas estrogen in females has been shown to exert protective effects through modulation of hepatic stellate cell activity and lipid metabolism (32, 33). These differences arise from hormonal influences, as well as genetic and epigenetic regulators, which contribute to sex-biased liver gene expression (34). In addition, accumulating evidence indicates that maternal environmental exposures—such as nutritional imbalance during gestation—can have sex-specific impacts on the offspring's metabolic programming and disease susceptibility (35). Therefore, to comprehensively evaluate the biological consequences of maternal high-salt diet exposure, this study included both male and female offspring. This approach facilitates the identification of sexually dimorphic responses and their implications for liver disease risk later in life.

To investigate the effects of an HSD on the structure of liver tissue in offspring mice, we developed an HSD model by feeding pregnant C57BL/6J mice with sodium-rich chow containing 4% NaCl. Using high-throughput RNA sequencing (RNA-seq) as a powerful tool for exploring alterations in gene expression, offering an innovative approach to uncovering specific biological processes and associated novel mechanisms (36), in the livers of offspring mice treated with HSD, we aim to uncover altered patterns in the entire gene transcriptome. Subsequently, through differential expression analysis and pathway enrichment analysis, we determined that HSD induces hepatic dysfunction and structural damage in offspring mice's livers by altering metabolism, immune response, fibrosis, and apoptosis, potentially leading to liver-related diseases. Therefore, our study elucidates alterations in transcriptomic gene expression in the liver tissues of offspring following maternal HSD ingestion, suggesting novel molecular-level mechanisms crucial for developing therapeutic interventions against HSD-induced liver-related diseases.

## 2 Materials and methods

### 2.1 Overall study design

The experimental design's schematic is presented in Supplementary Figure S1. Pregnant mice in the HSD group received 4% NaCl chow (equal to eight times more salt than a normal healthy diet) from confirmation of pregnancy (GD0) through lactation (P0-P21). Pregnant mice in the normal group received standard chow for the same period. At weaning (P21), all offspring continued on the same diet as their mothers until P28 (4-week-old offspring). Subsequently, body weight and liver weight data were collected from normal-treated male offspring mice (CONM), HSD-treated male offspring mice (HSDM), normal-treated female offspring mice (CONF), and HSD-treated female offspring mice (HSDF). Liver tissue was subjected to pathological staining, and transcriptional profiles were obtained using RNA sequencing (RNA-Seq). Finally, CMap analysis was performed to identify potential small-molecule compounds that could ameliorate liver damage in offspring.

### 2.2 Animals and tissue collection

Twenty-four C57BL/6J wild-type mice (containing 16 females and eight males) aged 6–8 weeks were purchased from GemPharmatech Co., Ltd (Nanjing, Jiangsu). The newly acquired C57BL/6J mice were acclimated to a 12-h light/12-h dark cycle for 2 weeks before being mated at a ratio of two males to one female. Males were removed from cages once females became pregnant. During the experiment, pregnant mice were randomly divided into two groups: the control group (containing six pregnant mice) and the HSD group (containing 10 pregnant mice). Each pregnant mouse was placed in a separate cage to exclude miscarriage, stillbirth, or other factors that occurred during pregnancy, and the normal production of offspring was used for follow-up experiments. Litter sizes were not standardized post-natally; to reduce bias, each dam was housed separately. Offspring with body weights outside ±2 SD of the group mean at weaning were excluded (to minimize potential confounding due to litter variability or intrauterine growth restriction), and equal numbers of males and females were randomly selected from different litters. Offspring at 4 weeks of age were euthanized by cervical dislocation, and liver tissues were collected and weighed in the CONM, HSDM, CONF, and HSDF groups (each group included nine offspring after weight-based exclusions). All animals were euthanized and liver tissues collected during the light phase of the 12:12 cycle (09:00–11:00 a.m.) to minimize diurnal variation in hepatic weight and gene expression. Subsequently, the tissues were immediately immersed in liquid nitrogen and stored at −80 °C in a freezer. These liver tissues were collected for subsequent pharmacological experiments and RNA sequencing. Specifically, nine male and nine female offspring were included in each of the following experimental groups: CONM, CONF, HSDM, and HSDF. Three animals per group were used for transcriptomic analysis, and six animals were used pathological evaluation and therapeutic intervention studies. All experiment procedures were conducted in accordance with ARRIVE guidelines and approved by the Institutional Animal Care and Use Committee of Henan Province People's Hospital, and shall be conducted in accordance with relevant regulations.

### 2.3 H&E staining of the mouse liver

Hematoxylin-eosin staining (H&E staining) is one of the most used methods for staining paraffin sections, confirming the fundamental cellular structure. All experimental procedures were conducted according to previously published methods (37, 38). Xylene and ethanol reagents were purchased from Sinopharm Chemical Reagent Co., Ltd. (Shanghai, China). The experimental steps were as follows: liver tissues from CONM, HSDM, CONF, and HSDF groups were embedded in paraffin and sectioned using a paraffin slicer. Subsequently, the liver sections were sequentially immersed in xylene I–III and dewaxed for 5 min each, followed by a series of anhydrous ethanol concentrations (100%−65%) for 1 min, and rinsed with distilled water. Next, the liver tissue sections were sequentially stained with hematoxylin and eosin staining solutions. Finally, the sections were dehydrated, immersed in xylene, dried, and sealed with neutral resin. The liver sections were observed, imaged, and analyzed using a Nikon ECLIPSE E100 light microscope (Japan). Twenty 400 × field images were selected, and the quantification of liver cell size was performed in a double-blind manner. Additionally, the number of cells in each field was counted using ImageJ software (United States). Average cell size was calculated as the number of cells divided by the area of the field.

### 2.4 Masson staining of the mouse liver

Masson staining is primarily utilized for evaluating tissue fibrosis. All experimental procedures were conducted according to previously published methods (39, 40). Paraffin embedding, sectioning, and deparaffinization were conducted following the same procedure as for H&E staining. The staining process proceeded as follows: (1) sections were stained with Weigert's iron hematoxylin solution for 5–10 min, followed by quick rinsing with distilled water to remove excess dye; differentiation was carried out using acidic ethanol differentiation solution and rinsed again with distilled water for 5 min. (2) Sections were stained with acid fuchsin solution of Ponceau for 5 min, followed by rinsing with weak acid solution for 1 min; differentiation was then performed using a 1% phosphomolybdic acid solution and washed with weak acid. (3) Aniline blue was applied and stained for 1 min, followed by washing with a weak acid. Subsequently, the sections were washed, dried, and sequentially sealed. The extent of fibrosis was assessed by microscopic observation and analysis of liver tissue sections. Twenty 400 × field images were randomly chosen to quantify the area of fibrotic tissue using a double-blind approach. Similarly, the ratio of fibrotic tissue area was determined using ImageJ software (United States).

### 2.5 Immunohistochemistry of mouse liver

Immunohistochemistry (IHC) is a technique that involves the binding of antigens to antibodies to visualize protein expression in structures and tissues. This experiment followed procedures described in the literature (38, 41, 42). After dewaxing, antigen retrieval, inactivation, sealing, incubation with primary and secondary antibodies, color development, restaining, and dehydration sealing, immunohistochemical images were obtained. The liver sections were observed, imaged, and analyzed using a light microscope (Pannoramic 250/MIDI, Hungary). Twenty 400 × field images were selected, and positive cells were counted in a double-blind manner to quantify the infiltration of F4/80 macrophages and apoptosis. Additionally, version 1.53k of ImageJ software, by Wayne Rasband (United States), was used to calculate the total number of cells.

### 2.6 RNA extraction and RNA sequencing

The liver tissues of the CONM, HSDM, CONF, and HSDF groups were collected, weighed, rapidly frozen in liquid nitrogen, and stored at −80 °C. First, frozen mouse liver tissues were ground using a mortar, and RNA was extracted using the RNA Easy Spin Column Kit (Qiagen, Germany) following the TRIzol RNA extraction protocol. Second, after qualifying the samples, RNA libraries were prepared using the DNBSEQ platform (43). The process involved: (1) enriching eukaryotic mRNA using magnetic beads with Oligo (dT), (2) fragmenting mRNA using the Fragmentation Buffer, and (3) using mRNA as a template to synthesize the first strand of complementary DNA (cDNA) with six-base random primers (random hexamers), followed by the addition of buffer, dNTPs, RNase H, and DNA polymerase I to synthesize the second strand of cDNA. The cDNA was purified using AMPure XP beads. (4) The purified double-stranded cDNA was subsequently end-repaired, A-tailed, ligated to sequencing adapters, and subjected to fragment size selection using AMPure XP beads. (5) The cDNA library was obtained by PCR enrichment. Third, after library construction, the library concentration and insert size were measured, and the effective concentration was quantified using q-PCR to ensure library quality. Finally, after confirming the quality of the library, high-throughput sequencing was conducted. RNA extraction and sequencing were performed at BGI (Shenzhen, China). The reference genome used in this study belongs to *Mus musculus*, derived from NCBI, the version was GCF_000001635.26_GRCm38.p6.

### 2.7 Quantitative real-time PCR (RT-qPCR)

Total RNA was extracted from frozen liver tissues using the Total RNA Extractor (TRIzol) Kit (Cat. No. B511311; Sangon Biotech, Shanghai, China) following the manufacturer's protocol. RNA concentration and purity were assessed via spectrophotometry prior to complementary DNA (cDNA) synthesis. cDNA was synthesized using the Maxima Reverse Transcriptase Kit (Cat. No. EP0743; Thermo Fisher Scientific) according to the manufacturer's instructions. RT-qPCR reactions were carried out using the SGExcel FastSYBR Mixture Kit (Cat. No. B532955; Sangon Biotech, Shanghai, China) under standard cycling conditions. Gene expression levels were normalized to the internal reference gene GAPDH, and relative expression was calculated using the comparative Ct (ΔΔCt) method. For validation of gene expression, mRNA levels of *CPT1a, ACOX2, CYP4A14, Xbp1, Per2*, and *CD68* in liver tissue were quantified as previously described (44–46), using the primer sequences as shown in Supplementary Table S1.

### 2.8 Differentially expressed genes

Differentially expressed genes (DEGs) are those whose expression levels change significantly under different conditions. The primary goal of transcriptomics is to identify genes that differ between different comparison groups, thereby elucidating the molecular mechanisms underlying these differences. Therefore, the analysis of differential gene expression is crucial for interpreting the results (47, 48). The threshold criterion for statistically significant DEGs is set at |log2FC| ≥1 and *Q*-value (or *P*-value) < 0.05. Additionally, genes across the entire transcriptome were classified based on their FPKM (fragments per kilobase million) expression levels: genes with low expression, 0 < FPKM < 1; genes with higher expression, 1 ≤ FPKM < 20; and genes with highly expressed, FPKM ≥ 20 (49).

### 2.9 Principal component analysis

Principal component analysis (PCA) is used to assess the correlation and dispersion among samples in sequencing data (50). As previously reported, PCA results were visualized using Statistical Analysis of Metagenomic Profiles (STAMP) software. In this study, PCA was used to illustrate the overall similarities or differences in the expression profiles of liver transcript genes among all offspring in the CONM, HSDM, CONF, and HSDF groups.

### 2.10 Functional enrichment GO and KEGG analyses

Gene Ontology (GO) functional enrichment analysis was conducted using BGI's *Dr. Tom* multi-omics interactive system to identify the primary molecular functions and biological processes associated with DEGs. GO terms meeting these criteria [*P* < 0.05 and false discovery rate (FDR) < 0.05] were considered statistically significance (38, 51). Metabolic and signal transduction pathways of DEGs were analyzed using Kyoto Encyclopedia of Genes and Genomes (KEGG) pathway enrichment. Pathways with a *P*-value of < 0.05 were considered significantly enriched with DEGs.

### 2.11 Gene set enrichment analysis

Gene Set Enrichment Analysis (GSEA) is a computational biology method used to analyze gene expression data with the aim of uncovering gene expression patterns associated with specific biological processes, pathways, or functions. Specifically, metabolic pathways in HSD-treated male and female offspring were analyzed using GSEA software version 4.0.3. Typically, pathways with |NES| >1, NOM *P*-value of < 0.05, and FDR *q*-value of < 0.25 are considered significantly enriched.

### 2.12 Protein–protein association networks

This study focused on revealing protein–protein association networks (PPANs) of metabolism-related, immune-related, fibrosis-related, and apoptosis-related genes. These results of PPANs were represented by the STRING database (https://string-db.org/) (52).

### 2.13 Identification of potential small-molecule drugs for the treatment of high salt diet-induced liver injury

The study utilized the Connectivity Map (CMap) online platform (https://clue.io/) to identify candidate small molecule drugs with inverse expression profiles to HSD-induced hepatic transcriptomic changes. Compounds with high fdr_q_nlog10 values and low norm_cs scores were prioritized due to their predicted ability to reverse key DEGs and pathways associated with HSD-induced liver injury.

Following weaning (at 4 weeks of age), offspring were randomly assigned to treatment or vehicle control groups (*n* = 6 mice per group per sex). Male offspring were treated with ketoprofen (1 mg/kg, oral gavage, three times per week for 4 weeks; MedChemExpress Co., Ltd), based on literature supporting its anti-inflammatory and anti-fibrotic efficacy in liver injury models with male-predominant pathology (53). Female offspring received intraperitoneal injections of BRD-K88741031 (1 mg/kg, three times per week for 4 weeks; MedChemExpress Co., Ltd), a PPARγ agonist known to modulate metabolic pathways, such as PPARA and NR1D1, that were disrupted in female offspring. (54, 55).

Sex- and diet-matched HSD-only groups receiving vehicle treatment served as negative controls. All animals were monitored throughout the treatment period for signs of overt toxicity, including changes in body weight, grooming behavior, and food intake. No visible signs of systemic toxicity were observed. At the end of the intervention, liver tissues were harvested for histopathological analysis. Molecular assays (e.g., post-treatment RNA-seq, RT-qPCR, and protein validation) were not performed in this study but are planned for future investigations.

### 2.14 Statistical analysis and software

The Venn diagram plotter (Venny) version 2.1.0 and Excel 2021 software were used to compare the transcript counts between the normal offspring group and the HSD-treated offspring group. PCA was performed using STAMP statistical software. Additionally, the heatmap was generated using R (64-bit, version 3.5.3). GraphPad Prism software version 8.1.0 (La Jolla, CA, USA) and SPSS 27.0 software (IBM) were used to generate bar, line, and scatter plots. A two-way analysis of variance (ANOVA) was performed. The data are presented as mean ± SEM (standard error) and were considered statistically significant at a *P*-value of < 0.05.

## 3 Results

### 3.1 Maternal HSD leads to liver injury in offspring mice

**Figure 1:**
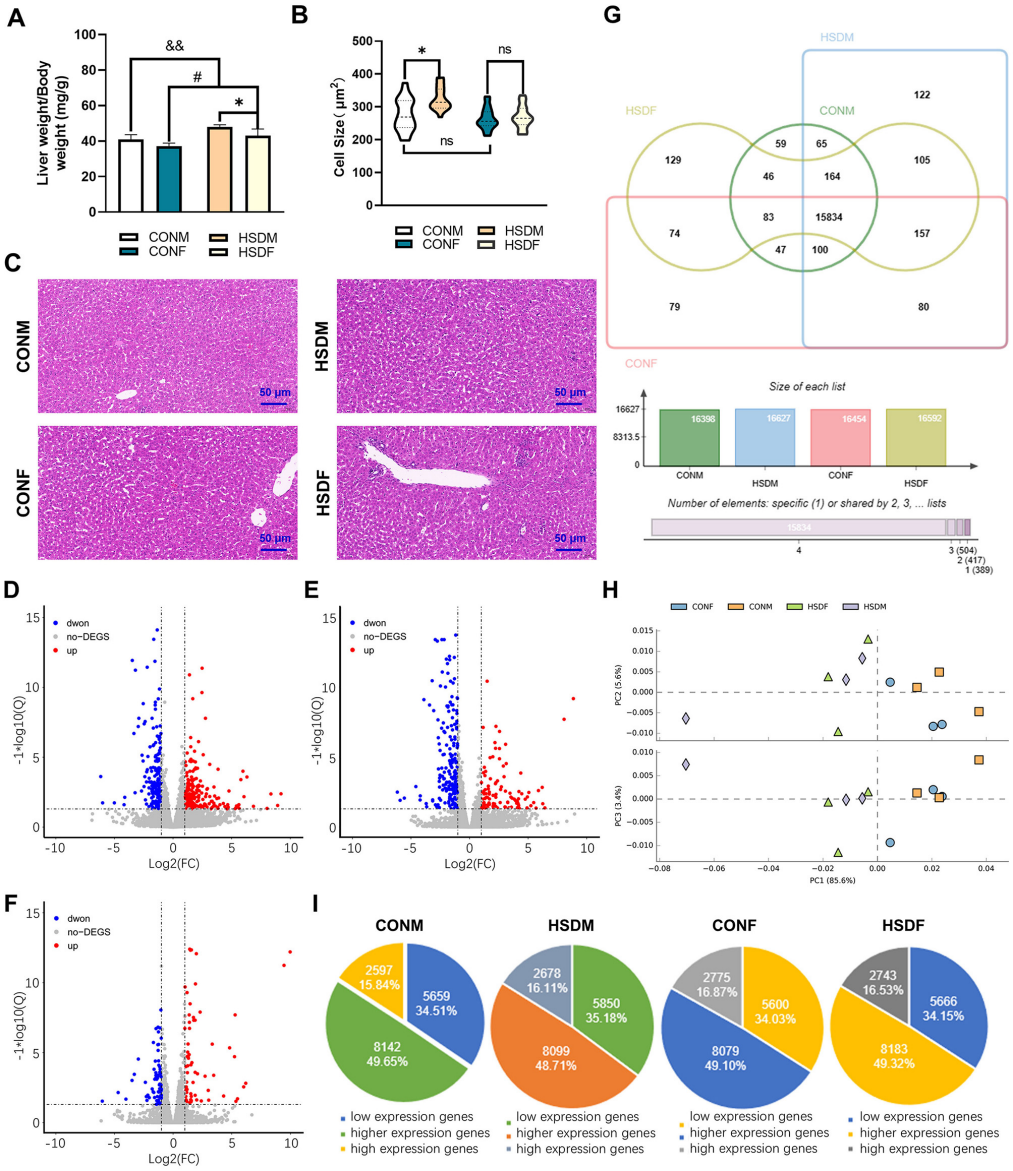
Effects of maternal HSD on the liver of male and female offspring in mice. **(A)** Ratio of liver weight to body weight of CONM, HSDM, CONF, and HSDF (*N* = 6 per group), ^*^P < 0.05, ^#^*P* < 0.05, ^&&^*P* < 0.01. **(B)** Liver cell size in CONM, HSDM, CONF, and HSDF (*N* = 6 per group), ^*^P < 0.05, ns: no significant difference. **(C)** Histopathology of the liver tissue from the male and female offspring in mice, representative micrographs of liver sections stained with H&E staining. The scale bar represents 50 μm. **(D–F)** Scatter plots displaying differentially expressed genes (DEGs) between control (CONM and CONF) and HSD-treated (HSDM and HSDF) groups. Red dots indicate upregulated genes, while green dots represent downregulated genes. Each plot compares gene expression levels within respective groups. **(G)** Venn diagrams illustrate the overlap and unique transcripts identified in the liver transcriptomes of offspring from normal and HSD-treated mice. **(H)** PCA scatter plot depicting gene expression patterns in the liver tissues of CONM (orange square), HSDM (pale purple diamond), CONF (blue dot), and HSDF (light green triangle) groups. The plot shows clustering and dispersion of gene expression profiles. **(I)** Pie charts showing the distribution of low, higher, and highly expressed genes in each experimental group (CONM, HSDM, CONF, and HSDF). The charts illustrate the proportion of genes categorized based on their expression levels. *N* = 3 mice per group.

This study aimed to determine whether an HSD affects the liver weight and structure of offspring mice by measuring body weight, liver tissue weight, and analyzing HE staining results of liver tissue (25, 56). The HSD increased the ratio of liver weight to body weight in newborn mice compared to the control group. A two-way ANOVA revealed a significant main effect of HSD (*P* = 0.001 and *P* = 0.014), but a small main effect of sex × HSD interaction (*P* = 0.022) on ratios of liver mass and body weight (Figure 1A). Liver cell size in male offspring of the HSD group was significantly larger than in the control group. However, no significant difference in liver cell size was observed between female mice treated with an HSD and the control group. Additionally, male mice in the control group exhibited slightly larger hepatocytes than female mice. ANOVA indicated a significant HSD interaction (*P* = 0.041) with male cell size (Figure 1B). H&E staining results corroborated these findings (Figure 1C). Overall, these results indicate that maternal HSD induced liver injury in the offspring mice.

### 3.2 Maternal HSD alters sex-specific transcriptome composition in the livers of mouse offspring

To investigate the impact of excessive maternal salt intake during pregnancy and lactation on liver transcriptome gene expression in offspring, we conducted transcriptomic analyses on liver tissues from male and female offspring. The Venn diagram and PCA results are presented below. In male mouse offspring, 16,398 and 16,627 transcripts were identified in the livers of the normal male (CONM) and HSD-treated male (HSDM) groups, respectively. Similarly, in female mouse offspring, 16,454 and 16,592 transcripts were detected in the normal female (CONF) and HSD-treated female (HSDF) livers, respectively. Notably, 229 genes in CONM and 138 genes in CONF were below the detection threshold (Figure 1G). Principal component analysis (PCA) revealed that PC1, PC2, and PC3 contributed 85.60%, 5.60%, and 3.40% of the total variance, respectively, illustrating intra-group aggregation and inter-group dispersion of genes between CONM and HSDM, as well as between CONF and HSDF (Figure 1H). Differentially expressed genes (DEGs) were visualized using scatter plots (Figures 1D–F). In male offspring, 466 DEGs were identified between CONM and HSDM, including 257 upregulated and 209 downregulated genes (Figure 1D and Supplementary Table S2). In female offspring, 309 DEGs were found between CONF and HSDF, with 102 upregulated and 207 downregulated genes (Figure 1E and Supplementary Table S3). Furthermore, we compared the number of DEGs between males and females in the normal group and found a total of 151 DEGs, including 78 upregulated and 73 downregulated genes, which were clearly different from the HSD-treated group (Figure 1F and Supplementary Table S4). To validate the RNA-seq findings, RT-qPCR analysis was performed on liver tissue to assess the expression of *CPT1a, ACOX2, CYP4A14, Xbp1, Per2*, and *CD68* (44, 45, 57–59). These genes were chosen for their roles in lipid metabolism, ER stress response, circadian regulation, and immune cell activation. The expression patterns observed in RT-qPCR were consistent with those in RNA-seq results for both male and female offspring (Supplementary Figure S2 and Supplementary Table S1), supporting the reliability and reproducibility of the transcriptomic analysis.

Finally, transcripts were classified into low, higher, and highly expressed genes, revealing that maternal HSD alters the liver transcriptome composition in offspring mice, particularly exhibiting gender-specific differences. In the CONM group, among the 16,398 transcripts, 5,659 (34.51%) were low expression genes, 8,142 (49.65%) were higher expression genes, and 2,597 (15.84%) were highly expression genes; and in HSDM group, the low, higher, and highly expression genes accounted for 5,850 (35.18%), 8,099 (48.71%), and 2,678 (16.11%) from 16,627 transcripts, respectively. However, for female offspring, among the 16,454 transcripts in the CONF group, 5,600 (34.03%) were low expression genes, 8,079 (49.10%) were higher expression genes, and 2,775 (16.87%) were highly expression genes, and from the 17,036 transcripts in the HSDF group, the low, higher, and highly expressed genes accounted for 5,100 (29.94%), 8,565 (50.28%), and 3,371 (19.79%), respectively (Figure 1I). The above results conclude that excessive salt intake by the mother during pregnancy and lactation changes the liver transcriptome composition in offspring mice, and these changes exhibit sexual dimorphism.

### 3.3 HSD affected KEGG pathways of higher expressed genes in the offspring

**Figure 2:**
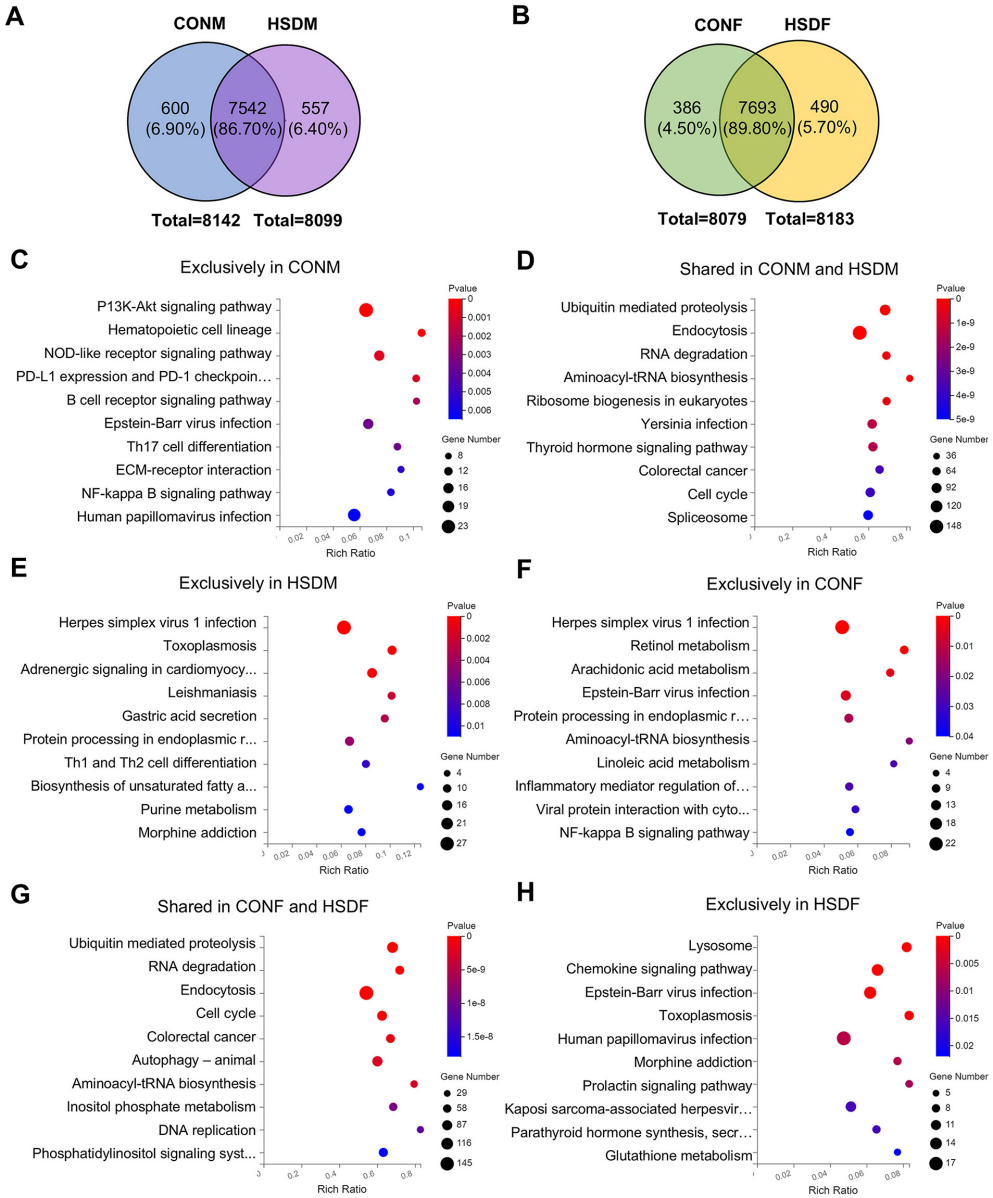
Maternal HSD alters KEGG pathways with sex-specific effects in the liver of offspring mice. **(A, B)** Venn diagram illustrating higher expression genes in the control group and HSD-treated group. **(C–E)** Annotation of the top 10 KEGG pathways enriched in higher expression genes between CONM and HSDM groups, with a *P*-value <0.05. **(F–H)** Annotation of the top 10 KEGG pathways enriched in higher expression genes between the CONF and HSDF groups, with *P* < 0.05. *N* = 3 mice per group.

To investigate the transcriptomic impact of maternal HSD on offspring liver, we focused on the higher expressed genes (main screening criteria: 1 ≤ FPKM < 20), which represent most of the gene expression. Venn diagrams and KEGG enrichment analyses were performed to compare expression patterns between CONM and HSDM, CONF and HSDF, and between sexes (Figures 2A, B and Supplementary Figure S3A). While changes in gene expression levels have been observed, pathway-level alterations remained unclear. Therefore, we further analyzed functional enrichment in both male and female offspring (Figures 2C–H and Supplementary Figures S3B–D).

In male offspring, 600 (6.90%) highly expressed genes were unique to the CONM group, 557 (6.40%) to the HSDM group, and 7,542 (86.70%) were shared (Figure 2A). KEGG analysis revealed 41 pathways enriched in CONM-specific genes, mainly involving the cell cycle, PI3K-Akt signaling, immune regulation, and various cancers (Figure 2C). The 7,542 shared genes were associated with 49 pathways, including autophagy, spliceosome, ubiquitin-mediated proteolysis, and cancer-related signaling (Figure 2D). The HSDM-specific genes were enriched in 36 pathways, notably endoplasmic reticulum processing, viral infections, metabolism, and Th1/Th2 differentiation (Figure 2E). Meanwhile, in female offspring, 386 (4.50%) genes were exclusive to the CONF group, 7,693 (89.80%) genes were shared between the CONF and HSDF groups, and 490 (5.70%) genes were exclusive to the HSDF group (Figure 2B). KEGG analysis showed that CONF-specific genes were enriched in 20 pathways, including autophagy, NF-kappa B, viral infections, and lipid metabolism (Figure 2F). The shared genes of the CONF and HSDF groups were associated with 146 pathways, such as cell cycle, ubiquitin-mediated proteolysis, and colorectal cancer (Figure 2G). HSDF-specific genes enriched 31 pathways, notably lysosome, chemokine signaling, glutathione metabolism, and several infections (Figure 2H). Additionally, as shown in Supplementary Figure S3A, 410 (4.80%) genes were exclusive to the CONM group, 7,732 (91.10%) genes were shared between the CONM and CONF groups, and 347 (4.10%) genes were exclusive to the CONF group. KEGG analysis revealed that CONM-specific genes were involved in 32 pathways related to viral infections, immune signaling, and glycan biosynthesis (Supplementary Figure S3B). Their 7,732 shared genes were enriched in 149 pathways, including endocytosis, autophagy, and colorectal cancer (Supplementary Figure S3C). In contrast, CONF-specific genes were enriched in only six pathways, mainly viral infections and mTOR signaling (Supplementary Figure S3D).

These findings reveal distinct transcriptomic responses to maternal high-salt intake between male and female offspring, characterized by both shared and sex-specific alterations in molecular pathways. While commonly affected pathways suggest core disruptions in cell cycle control and immune regulation, unique enrichment patterns in each group underscore sex-dependent sensitivities to metabolic, signaling, and infection-related processes. This highlights the importance of considering sex as a biological variable in evaluating developmental programming effects of prenatal environmental exposures.

### 3.4 HSD during pregnancy and lactation induces liver disease in offspring mice

**Figure 3:**
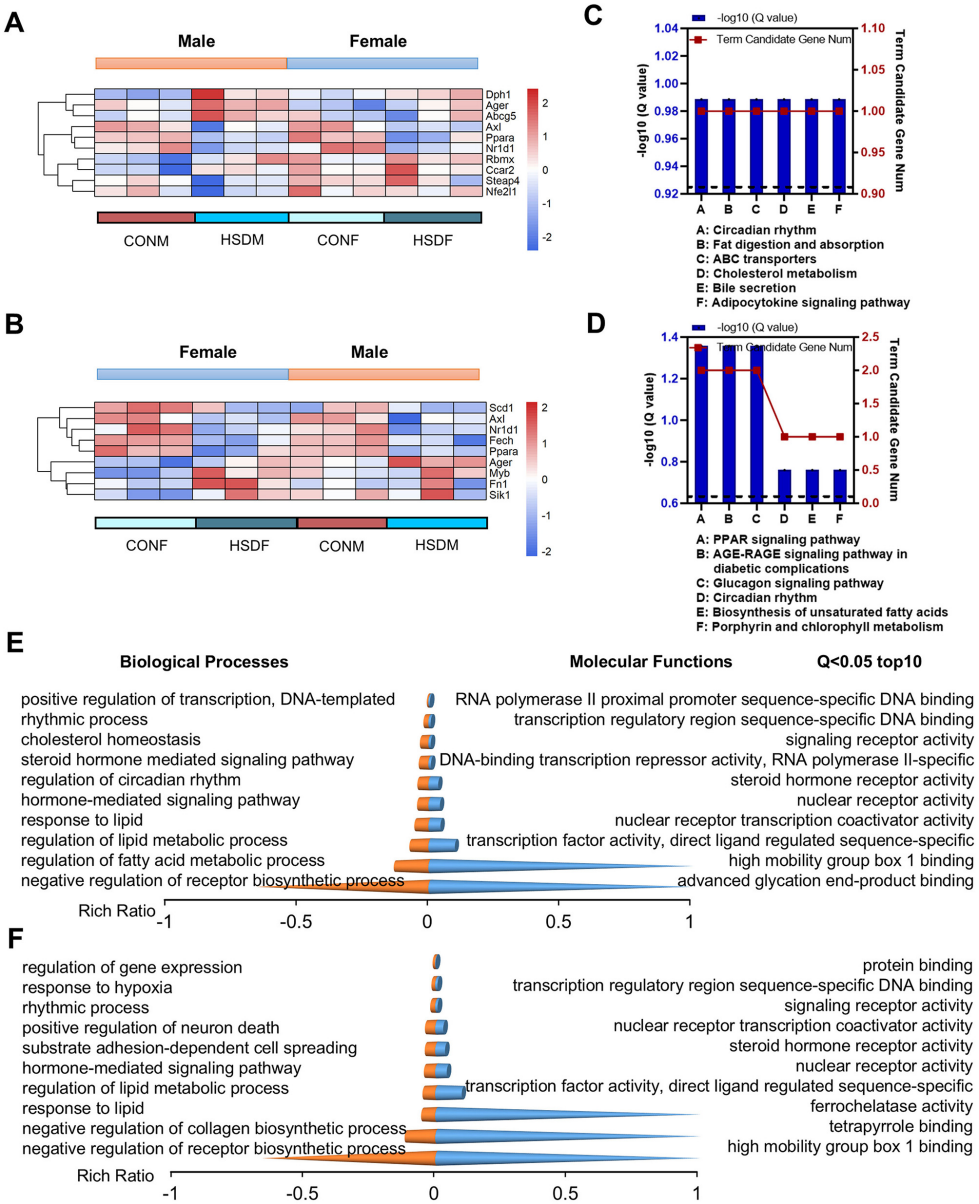
Maternal HSD induces changes in the expression profiles of liver-related genes and leads to varying degrees of liver disease in offspring mice. **(A, B)** Heatmap shows DEGs associated with liver diseases in male and female mouse offspring. **(C, D)** KEGG enrichment analysis of DEGs associated with liver diseases in male and female mouse offspring, with statistical significance at a *P*-value of <0.05. **(E, F)** Top 10 biological processes and molecular functions of DEGs associated with liver diseases in male and female mouse offspring, with statistical significance at *Q* < 0.05. *N* = 3 mice per group.

Usually, a high-salt diet induces various diseases, including cardiovascular and neurological diseases, as well as other organ lesions (19, 60, 61). Therefore, we investigated whether maternal HSD during pregnancy and lactation could induce liver-related diseases in offspring by examining the expression levels of differential genes in male and female offspring mice associated with liver diseases. As shown in the heat map, male offspring exposed to HSD exhibited differential expression of ten genes associated with liver disease compared to normal offspring: *Ager, Dph1, Steap4, Nfe2l1, Ppara, Rbmx, Nr1d1, Ccar2, Axl*, and *Abcg5* (Figure 3A). Conversely, female offspring showed differential expression of nine genes: *Ager, Fech, Fn1, Sik1, Myb, Ppara, Scd1, Nr1d1*, and *Axl* (Figure 3B). Furthermore, the KEGG pathways of liver disease-related genes in offspring mice were analyzed and found to be significantly different between male and female offspring. In male offspring mice, KEGG enrichment analysis revealed significant pathways related to liver diseases, including cholesterol metabolism (*P* = 3.00E-02) and fat digestion and absorption (*P* = 3.00E-02; Figure 3C). Conversely, for female offspring mice, KEGG enrichment analysis highlighted pathways related to liver diseases, such as biosynthesis of unsaturated fatty acids (*P* = 3.00E-02; Figure 3D).

Subsequently, we conducted an enrichment analysis of transcripts for GO molecular functions and biological processes associated with liver disease in male and female offspring. In male offspring, the molecular function can be classed into three categories (*P* < 0.05, the top 10 pathways are shown): (1) binding: RNA polymerase II proximal promoter sequence-specific DNA binding (*P* = 2.22E-04), high mobility group box 1 binding (*P* = 4.92E-04), advanced glycation end-product binding (*P* = 4.92E-04), transcription regulatory region sequence-specific DNA binding (*P* = 5.11E-04); (2) molecular transducer activity: steroid hormone receptor activity (*P* = 3.08E-04), signaling receptor activity (*P* = 4.77E-04), (3) transcription regulator activity: nuclear receptor transcription coactivator activity (*P* = 3.87E-06); transcription factor activity, direct ligand regulated sequence-specific DNA binding (*P* = 4.12E-05);, nuclear receptor activity (*P* = 2.23E-04), DNA-binding transcription repressor activity, RNA polymerase II-specific (*P* = 3.54E-04), while, the biological process can be classed into four categories (*P* < 0.05, the top 10 pathways are shown): (1) biological regulation: negative regulation of receptor biosynthetic process (*P* = 6.38E-07), cholesterol homeostasis (*P* = 1.02E-05),; regulation of lipid metabolic process (*P* = 8.74E-07), regulation of fatty acid metabolic process (*P* = 2.23E-05); positive regulation of transcription, hormone-mediated signaling pathway (*P* = 2.18E-04), DNA-templated (*P* = 2.02E-04);steroid hormone mediated signaling pathway (*P* = 3.01E-04); (2) regulation of biological process: regulation of circadian rhythm (*P* = 4.65E-06); (3) response to stimulus: response to lipid (*P* = 1.40E-04); and (4) rhythmic process: rhythmic process (*P* = 5.13E-05; Figure 3E and Supplementary Tables S5, S6).

For female offspring, the molecular function can be classed into four categories (*P* < 0.05, the top 10 pathways are shown): (1) binding: protein binding (*P* = 1.36E-04), transcription regulatory region sequence-specific DNA binding (*P* = 3.62E-04), tetrapyrrole binding (*P* = 4.43E-04), high mobility group box 1 binding (*P* = 4.43E-04); (2) catalytic activity: ferrochelatase activity (*P* = 4.43E-04); (3) molecular transducer activity: steroid hormone receptor activity (*P* = 2.47E-04) and signaling receptor activity (*P* = 3.38E-04); (4) transcription regulator activity: transcription factor activity, direct ligand regulated sequence-specific DNA binding (*P* = 3.30E-05), nuclear receptor activity (*P* = 1.79E-04), nuclear receptor transcription coactivator activity (*P* = 3.69E-04), while, the biological process can be classed into five categories (*P* < 0.05, the top 10 pathways are shown): (1) biological adhesion: substrate adhesion-dependent cell spreading (*P* = 2.06E-04); (2) biological regulation: negative regulation of receptor biosynthetic process (*P* = 5.11E-07), regulation of gene expression (*P* = 1.88E-05), regulation of lipid metabolic process (*P* = 1.38E-04); hormone-mediated signaling pathway (*P* = 1.75E-04), negative regulation of collagen biosynthetic process (*P* = 2.31E-05); (3) cellular process: positive regulation of neuron death (*P* = 2.15E-04); (4) response to stimulus: response to hypoxia (*P* = 8.31E-05); response to lipid (*P* = 1.13E-04); and (5) rhythmic process: rhythmic process (*P* = 3.61E-05; Figure 3F and Supplementary Tables S7, S8). In conclusion, maternal HSD during pregnancy and lactation exerts significant effects on the transcriptome composition of progeny livers, as indicated by differential gene expression and pathway analyses in both male and female mice.

### 3.5 HSD during pregnancy and lactation increases fibrosis in mouse offspring's liver

**Figure 4:**
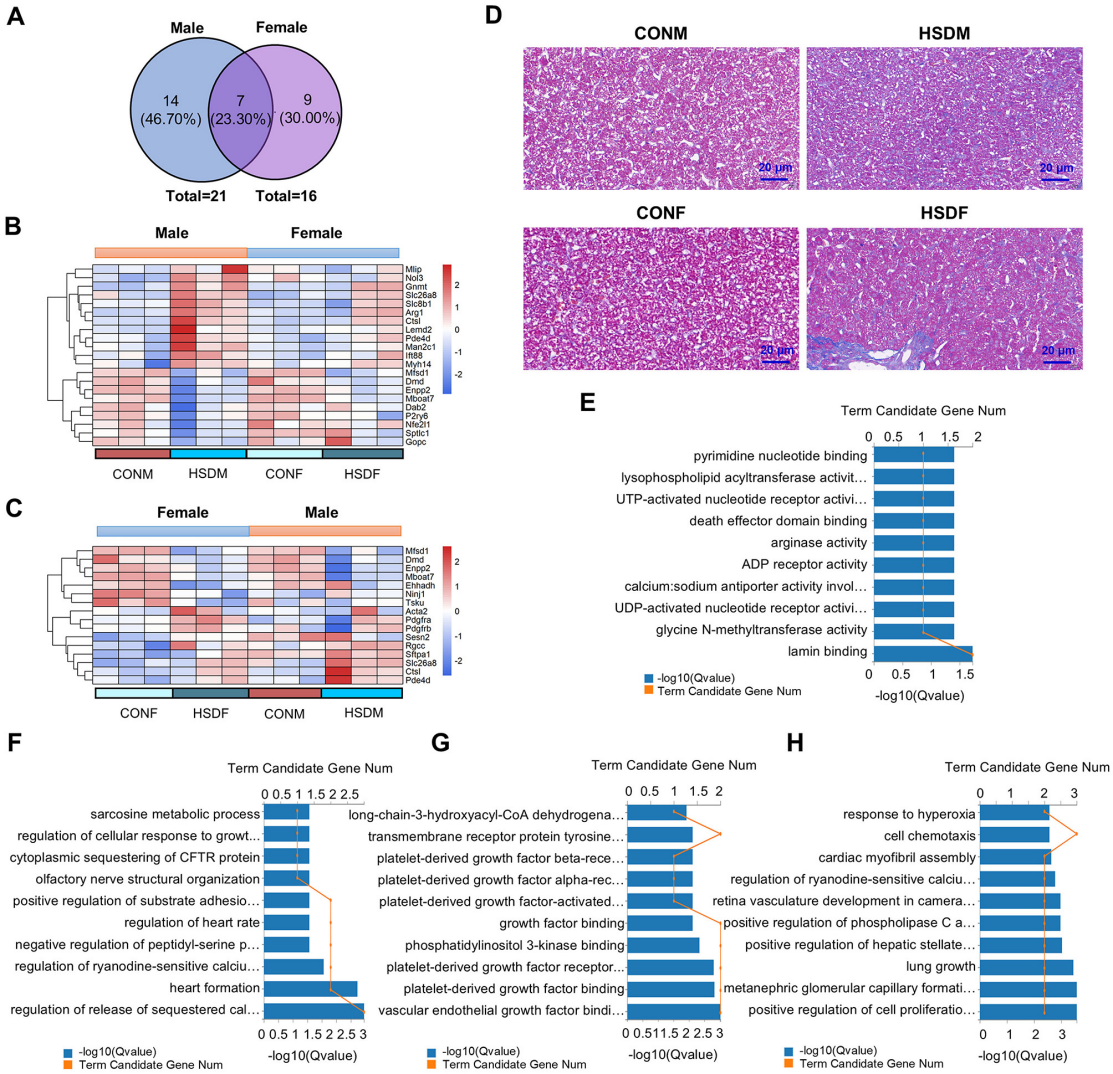
Maternal HSD alters the expression profile of liver fibrosis genes in offspring mice. **(A)** Venn diagram depicts liver fibrosis-related genes in male and female mice. **(B, C)** Heatmap shows DEGs associated with liver fibrosis in male and female mouse offspring. **(D)** Masson's staining of liver tissue obtained from CONM, HSDM, CONF, and HSDF. The scale bar represents 20 μm. **(E–H)** GO enrichment analysis (molecular functions and biological processes) of DEGs associated with liver disease in male and female mouse offspring, with the top 10 displayed in the figures, *Q* < 0.05. *N* = 3 mice per group.

Excessive intake of salt has been demonstrated in clinical studies to damage liver function and induce liver fibrosis (61). Therefore, this study aimed to investigate the effects of a maternal HSD on molecular and biological features associated with liver fibrosis in mouse offspring by analyzing differences in fibrosis-related transcript levels in their livers. Figure 4A illustrates that in the HSD-treated group, there were 21 significant differences in fibrosis-related transcripts in males and 16 in females. Specifically, 14 genes were exclusive to males, seven were shared, and nine were exclusive to females (46.70%, 23.30%, and 30.00%, respectively). Moreover, the heatmap indicated elevated gene expression associated with liver disease in offspring of mice from mothers on an HSD compared to controls, particularly noticeable in male offspring (Figures 4B, C). Genes related to liver fibrosis in both male and female offspring of the treatment group were further analyzed for molecular functions and biological processes. Among male offspring, there were 67 entries in molecular functions and 272 in biological processes (*P* < 0.05), while among female offspring, there were 57 entries in molecular functions and 239 in biological processes (*P* < 0.05). In conclusion, maternal HSD could influence the molecular functions and biological processes of fibrosis-related genes in offspring mice, albeit with differing effects (Figures 4E–H). Immunohistochemical results were utilized to investigate whether maternal HSD could accelerate liver fibrosis development in offspring. Masson's staining revealed that compared to the control group, male and female offspring subjected to HSD exhibited significantly increased liver fibrosis (*P* < 0.05; Figure 4D). Collectively, the heatmap, GO enrichment, and Masson's staining results suggested that maternal HSD tended to induce liver fibrosis in offspring mice.

### 3.6 HSD changes the expression levels of metabolism-related genes in the liver of offspring mice

Studies have shown that adverse maternal factors during pregnancy significantly impact offspring, leading to metabolic disorders (62, 63). In this study, we compared the expression levels of metabolism-related genes between male and female offspring groups to analyze changes in metabolic processes. In males, 423 genes showed significant differences in metabolism-related transcripts, compared to 252 in females. Of these, 266 genes (51.40%) were exclusive to males, 157 (30.30%) were shared, and 95 (18.30%) were exclusive to females (Supplementary Figure S4A). Additionally, KEGG enrichment analysis revealed significant differences in pathways. In male offspring, identified top 10 pathways included carbon metabolism (*P* = 3.63E-24), alanine, aspartate and glutamate metabolism (*P* = 3.35E-14), chemical carcinogenesis (*P* = 7.24E-17), retinol metabolism (*P* = 1.72E-15), biosynthesis of amino acids (*P* = 2.55E-23), fatty acid metabolism (*P* = 4.71E-14), metabolism of xenobiotics by cytochrome P450 (*P* = 4.24E-14), fatty acid degradation (*P* = 6.74E-14), valine, leucine and isoleucine degradation (*P* = 7.26E-14), and arginine biosynthesis (*P* = 2.01E-13; Supplementary Figure S4B). For female offspring, top 10 pathways included retinol metabolism (*P* = 1.26E-13), chemical carcinogenesis (*P* = 3.14E-13), steroid hormone biosynthesis (*P* = 6.73E-13), biosynthesis of unsaturated fatty acids (*P* = 2.14E-12), metabolism of xenobiotics by cytochrome P450 (*P* = 5.62E-12), purine metabolism (*P* = 8.68E-12), drug metabolism-cytochrome P450 (*P* = 9.43E-12), fatty acid metabolism (*P* = 1.39E-11), carbon metabolism (*P* = 3.71E-11), and fatty acid elongation (*P* = 2.73E-10; Supplementary Figure S4C). Subsequent GO enrichment analysis indicated that maternal HSD could influence the molecular functions and biological processes of metabolism-related genes in male and female offspring mice (Supplementary Figures S4D, E). Furthermore, GSEA analysis revealed significant enrichment of metabolic dysfunction in both male and female offspring, particularly pronounced effect in females. Among male offspring mice, only six metabolic pathways showed significant enrichment, including ribosome (NES = 1.92, P = 1.10E-02, FDR = 0.23; Supplementary Figure S4F). In contrast, 52 metabolic pathways were enriched in female offspring mice, such as endocrine and other factor-regulated calcium reabsorption (NES = 2.05, P = 1.11E-02, FDR = 0.005; Supplementary Figure S4G). In summary, these findings indicate that maternal HSD modifies the metabolic expression profile of the offspring mouse liver and accelerates metabolic disruption.

### 3.7 HSD increases biomarkers of inflammation in the liver in offspring mice

**Figure 5:**
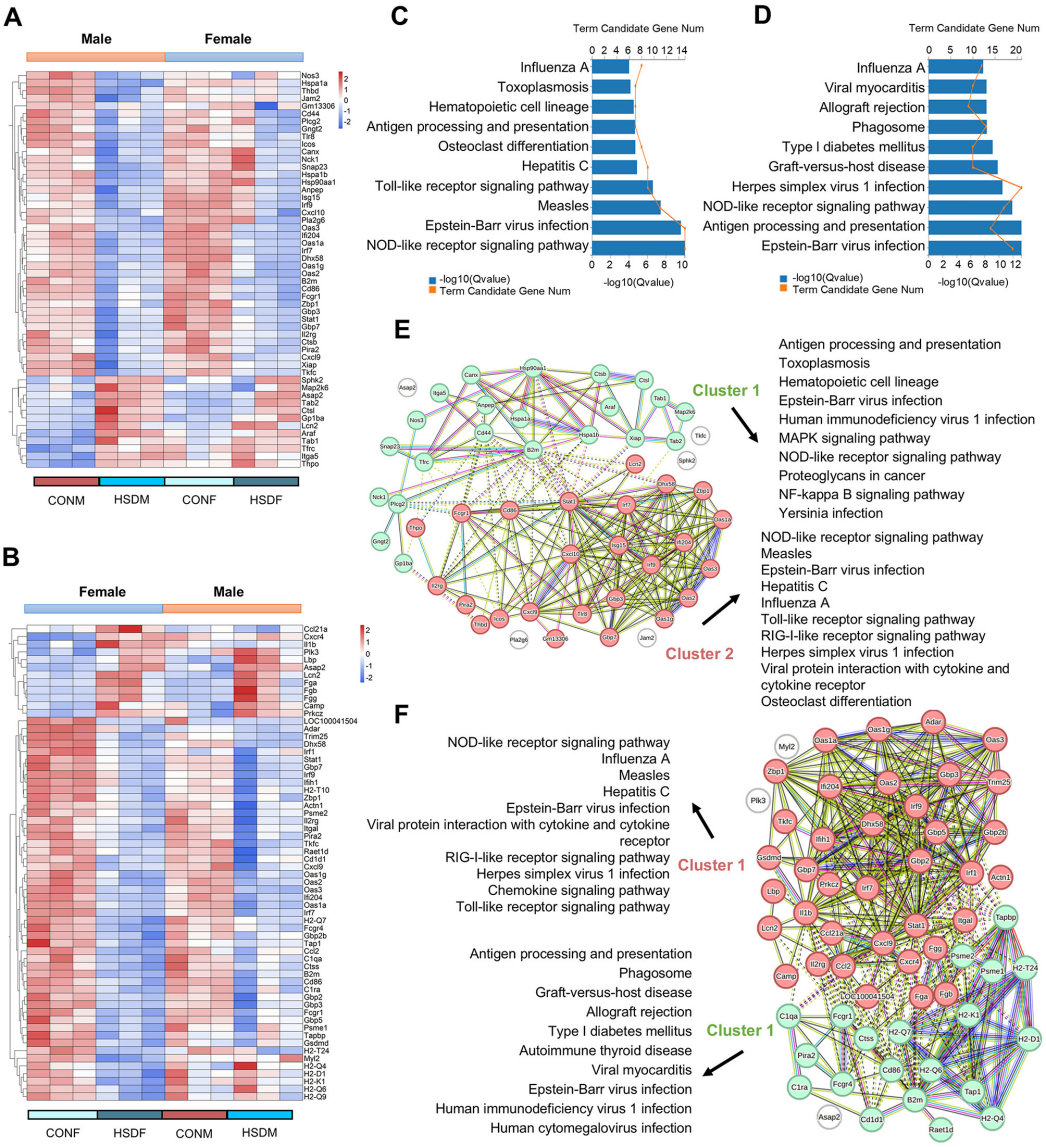
Maternal HSD leads to changes in the expression profile of liver immune-related genes of offspring mice. **(A, B)** Heatmap depicting liver immune-related DEGs in male and female mouse offspring. **(C, D)** KEGG enrichment analysis of liver immune-related DEGs in male and female mouse offspring, *P* < 0.05. **(E, F)** PPINs illustrate interactions and the top 10 enriched KEGG pathways associated with immunity-related genes in the liver of male and female offspring. *N* = 3 mice per group.

**Figure 6:**
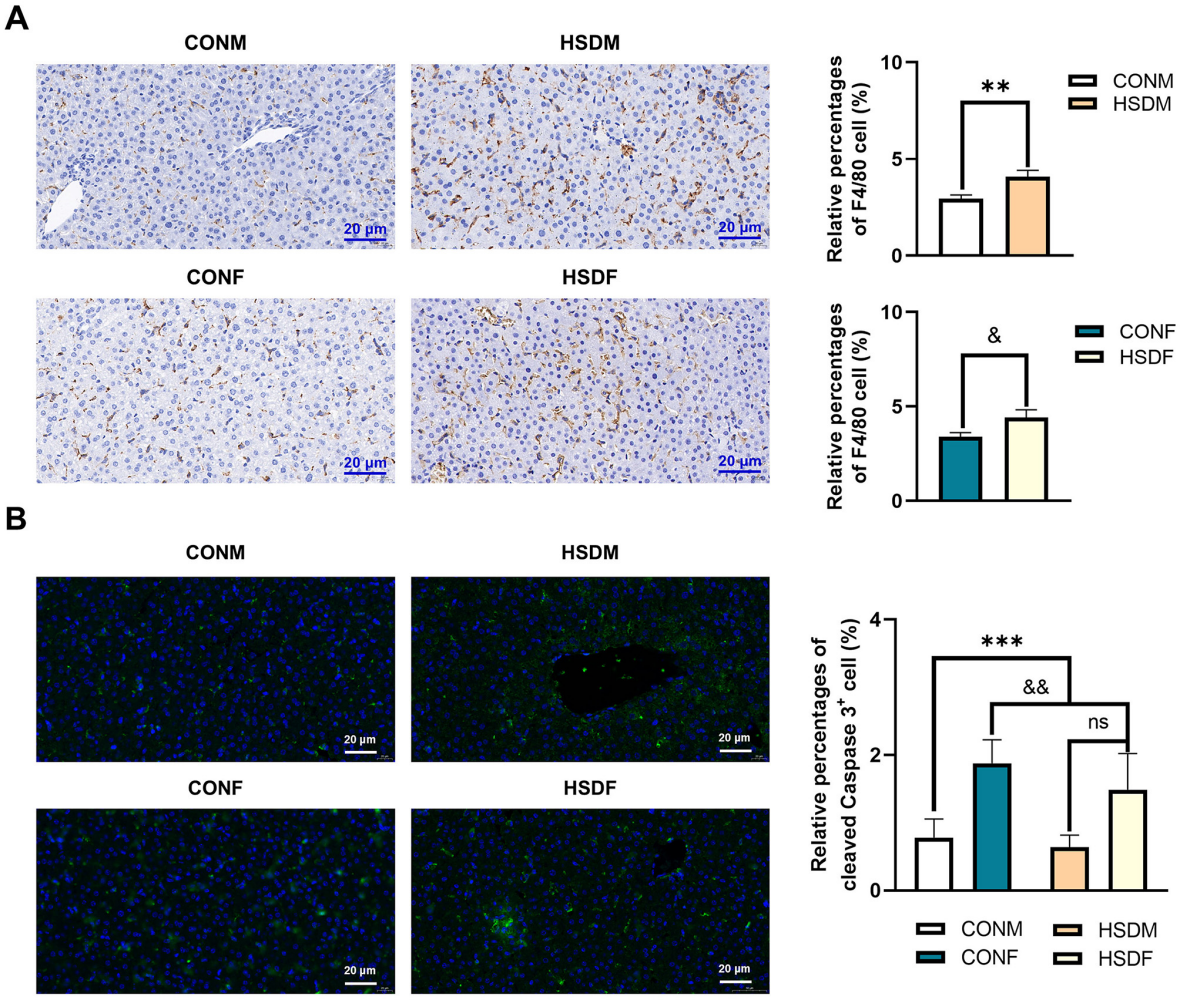
Effect of HSD on immunohistochemical and immunofluorescence staining in male and female mouse offspring. **(A)** Immunohistochemical staining showing F4/80-positive macrophages in liver tissue of normal and HSD-treated male and female mouse offspring. The scale bar represents 20 μm. Average relative abundance of F4/80-positive macrophages in liver tissue of normal and HSD-treated male and female mouse offspring (*N* = 6 per group). Statistical significance: ^**^P < 0.01, ^&^*P* < 0.05. **(B)** Immunofluorescence staining for cleaved caspase-3 in liver sections of normal and HSD-treated male and female offspring mice (*N* = 6 per group). The scale bar represents 20 μm. Statistical significance: ^&&^*P* < 0.01, ^***^P < 0.001, ns: no significant difference.

To investigate the impact of maternal HSD on immune-related molecular features in the offspring liver, we analyzed changes in immune-related transcript levels. In male offspring, 52 differential liver immune-related genes were identified between those treated with HSD and those on a normal diet (Figure 5A), while 62 such genes were identified in female offspring(Figure 5B). Subsequent KEGG enrichment analysis revealed significant pathway differences between males (49 pathways identified, *P* < 0.05) and females (57 pathways identified, *P* < 0.05). Figures 5C, D depict the top 10 enriched KEGG pathways for males and females, respectively. Using PPINs, we classified the complex interactions of differential immune-related genes in both sexes and illustrated their KEGG pathway distribution in Figures 5E, F. In males, interactions were divided into two clusters, with cluster 1 enriched in 57 pathways and cluster 2 in 26 pathways (*P* < 0.05), including pathways such as antigen processing and presentation (*P* = 1.52E-09) and the NOD-like receptor signaling pathway (*P* = 1.06E-10; Figure 5E). Similarly, in females, interactions formed two clusters, with 35 pathways enriched in cluster 1 and 29 in cluster 2 (*P* < 0.05), including pathways such as the chemokine signaling pathway (*P* = 1.86E-05) and antigen processing and presentation (*P* = 8.32E-20; Figure 5F). Furthermore, immunohistochemical staining revealed increased accumulation of F4/80-positive macrophages in the liver tissue of male and female offspring exposed to maternal HSD compared to controls (Figure 6A). These findings underscore that maternal HSD modifies liver immune-related gene expression and induces immune-inflammatory responses in offspring mice.

### 3.8 Maternal HSD promotes the apoptosis of liver cells in offspring mice

In this study, we demonstrated that a maternal HSD alters the expression of apoptosis-related transcriptome genes in the offspring liver, potentially accelerating liver cell apoptosis. Cleaved caspase-3 serves as an apoptosis marker. Consequently, we employed immunofluorescence staining to examine the impact of maternal HSD on liver cell apoptosis in offspring mice. Maternal HSD induced apoptosis in both male and female offspring mice compared to the control group. Additionally, there was a significant increase in cleaved caspase-3 expression levels in the liver of offspring exposed to HSD conditions (Figure 6B). The heatmap revealed significant differences in apoptosis-related transcript levels between male and female offspring liver tissues exposed to a maternal HSD during pregnancy and lactation, with 54 and 42 differentially expressed genes, respectively, compared to those of offspring from mothers on a normal diet (Supplementary Figures S5A, B). Of these, 37 genes (46.80%) were exclusive to males, 17 genes (21.50%) were shared between males and females, and 25 genes (31.60%) were exclusive to females (Supplementary Figure S5C). Furthermore, GO enrichment analysis indicated that maternal HSD alters the molecular functions and biological processes of apoptosis-related genes in male and female offspring mice, as depicted in the top 10 pathways (Supplementary Figures S5D–G). Overall, these findings indicate that maternal HSD has divergent effects on liver cell apoptosis in male and female offspring.

### 3.9 Screening treatment of high salt diet-induced liver injury: potential small molecule compounds

**Figure 7:**
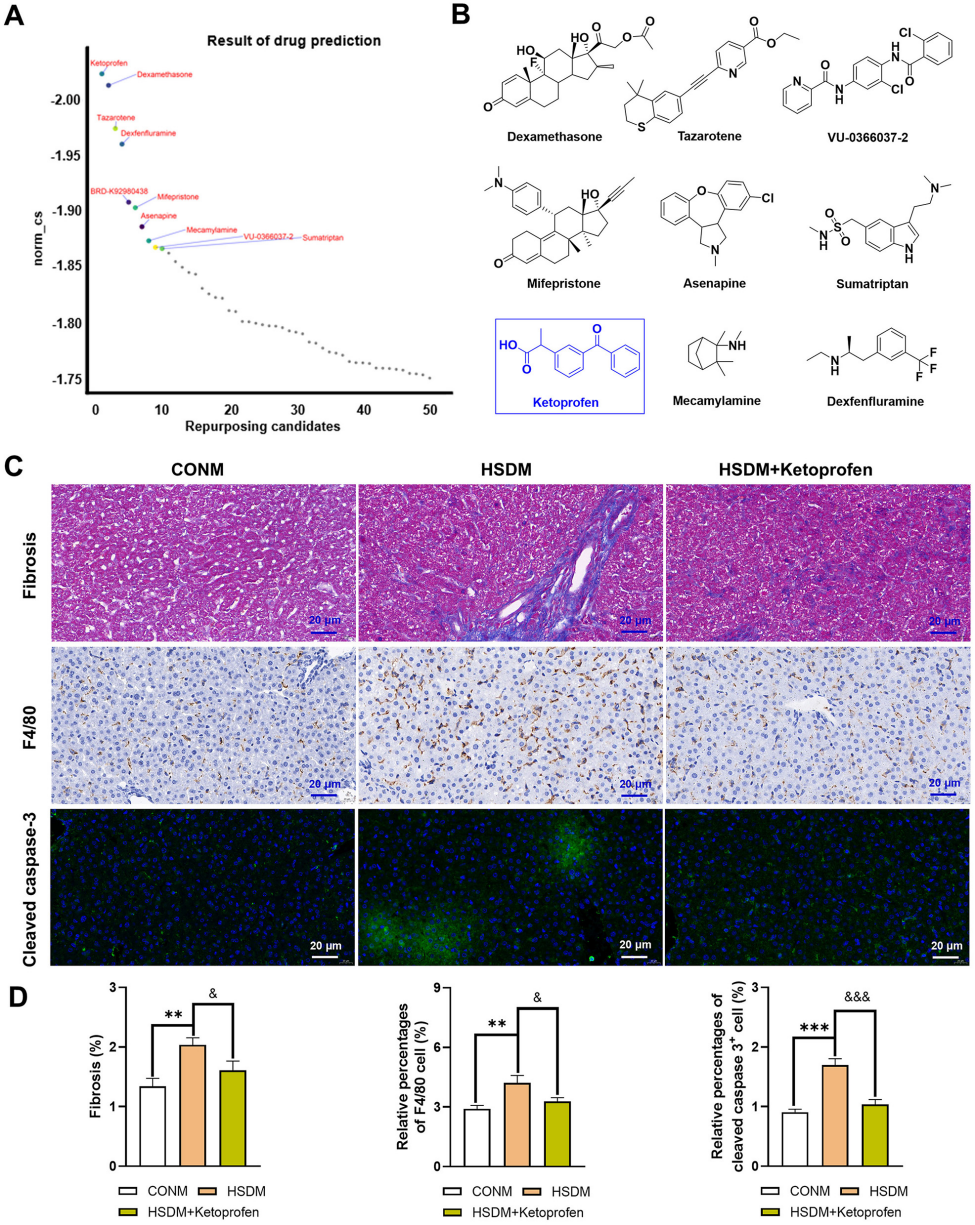
Potential small molecule compounds for the treatment of HSD induced liver injury in male offspring. **(A)** The top 10 compounds obtained by CMap analysis in male offspring. **(B)** Structural formula for small molecular compounds. **(C, D)** Staining for fibrosis, F4/80, and cleaved caspase-3 in liver sections of normal and HSD-treated male offspring mice. The scale bar represents 20 μm. Statistical significance: ^&^*P* < 0.05, ^**^P < 0.01, ^&*&&*^*P* < 0.001, ^***^P < 0.001. *N* = 6 mice per group.

**Figure 8:**
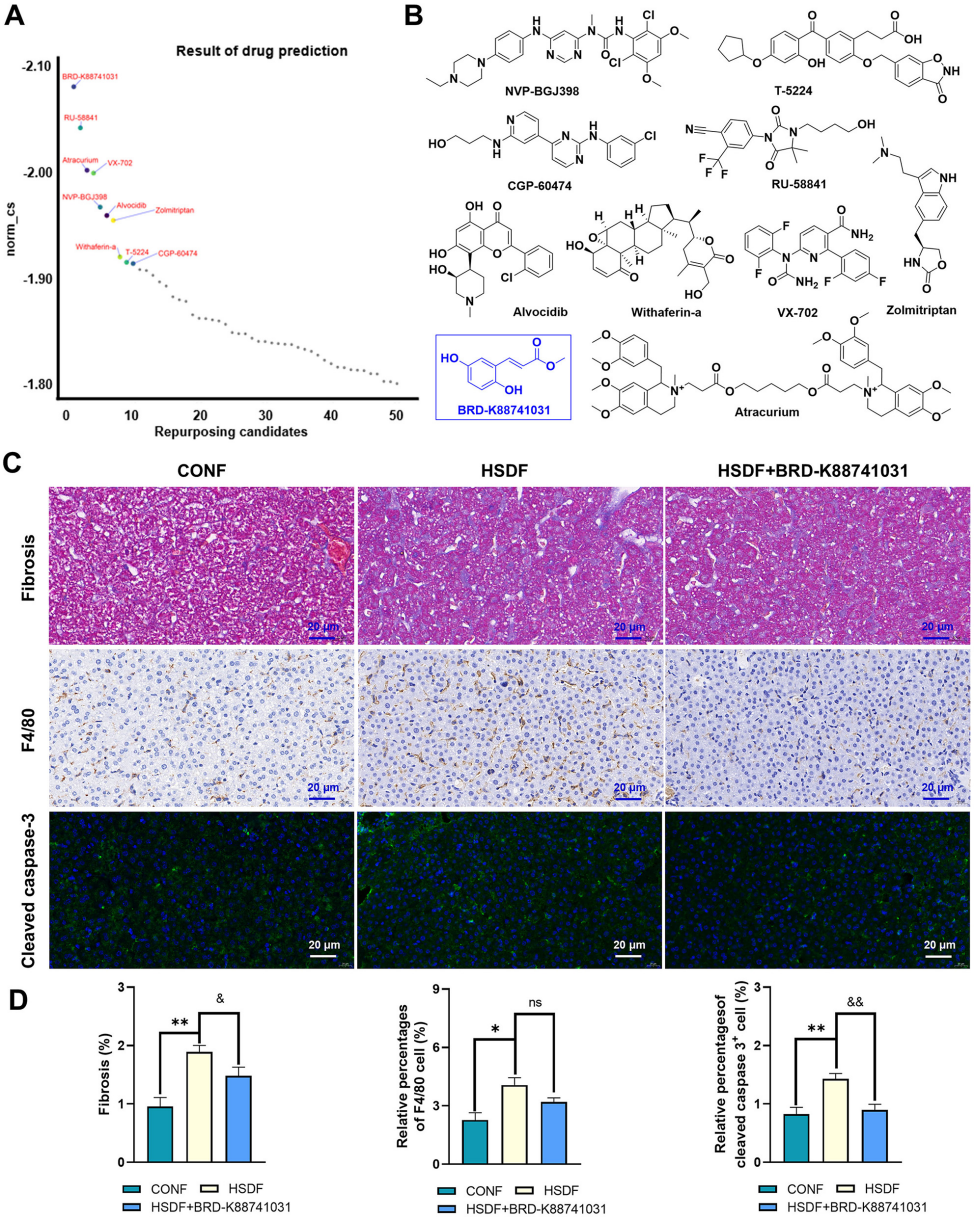
Potential small molecule compounds for the treatment of HSD induced liver injury in female offspring. **(A)** The top 10 compounds obtained by CMap analysis in female offspring. **(B)** Structural formula for small molecular compounds. **(C, D)** Staining for fibrosis, F4/80, and cleaved caspase-3 in liver sections of normal and HSD-treated female offspring mice. The scale bar represents 20 μm. Statistical significance: ^&^*P* < 0.05, ^*^P < 0.05, ^&&^*P* < 0.05, ^**^P < 0.01, ns: no significant difference. *N* = 6 mice per group.

CMap analysis was performed on the identified upregulated DEGs in male and female offspring mice, and the critical Top50 compounds with norm_cs < 0 in the results were selected (Supplementary Tables S9, S10), where the top 10 compounds were focused (Figures 7A, 8A). Among the top 10 compounds, according to a literature review, some small-molecule drugs, such as Ketoprofen (64, 65), dexamethasone (66–69), BRD-K88741031 (70), VX-702 (71, 72), have been reported to have significant fibrotic, anti-inflammatory, or apoptosis-inhibiting effects. In addition, ketoprofen reduces male-predominant fibrosis in NASH models, while PPARγ agonists rescue metabolic dysfunction in females. However, these compounds have not been studied for the treatment of liver fibrosis, immune infiltration, and apoptosis caused by a high-salt diet. To provide direct experimental evidence, we purchased ketoprofen (Figure 7B) and BRD-K88741031 (Figure 8B), respectively, to verify whether it has significant fibrotic, anti-inflammatory, or apoptotic effects in the livers of male and female offspring mice, respectively. The results showed that ketoprofen significantly improved the degree of fibrosis, cell infiltration, and apoptosis of male offspring mice treated with a high salt diet (Figure 7C). While each result was statistically significant (*P* < 0.05, Figure 7D). BRD-K88741031 significantly improved the degree of fibrosis, cell infiltration, and apoptosis in female offspring mice treated with a high-salt diet (Figure 8C). Furthermore, statistical differences for each item are shown in Figure 8D (*P* < 0.05). As a result, these pathological results confirmed that ketoprofen and BRD-K88741031 the HSD male and female offspring liver damage potential therapeutic effect.

## 4 Discussion

Salt is essential in daily human life, but excessive consumption can harm health. Inadequate maternal dietary habits during pregnancy and lactation may result in health issues for newborns. A significant finding of this study is that maternal HSD adversely affects the livers of offspring mice and can induce sex-specific liver diseases. Separate statements are as follows: (1) a maternal HSD had different effects on the body weight, liver weight, and liver structure of male and female offspring mice. (2) Maternal HSD altered the transcriptome composition of offspring liver tissue, leading to differential expression of genes and KEGG and GO-enriched pathways, with sex-specific differences. This finding is consistent with previous reports that organ transcriptome compositions are sex-specific (38, 73, 74). (3) Maternal HSD adversely affects the livers of offspring mice by increasing the infiltration of immunopositive cells, causing metabolic disorders, and enhancing liver fibrosis and cell apoptosis. Therefore, maternal exposure to adverse factors has an important impact on the health of the offspring. The results of this study also contribute to our understanding of the negative effects of a high-salt maternal diet on the livers of both male and female offspring.

Perinatal maternal lifestyle and environmental exposures have varied effects on offspring, including body weight and organ development. A high-fat diet during pregnancy can affect fetal body mass by enhancing the placental nutrient transport capacity (75). Exposure to higher concentrations of persistent organic pollutants, such as PM2.5, has been positively linked to macrosomia occurrence (76). In this study, we observed that an HSD during pregnancy and lactation led to offspring weight loss and altered the size of liver cells in the offspring mice. This study found sex differences in liver transcriptome composition in both male and female offspring. The number of differentially expressed genes in male offspring was approximately 1.16 times higher than in female offspring. Additionally, the number and types of highly expressed genes differed between male and female offspring. RNA-seq further revealed sex-dependent alterations in genes related to fibrosis, inflammation, and apoptosis, suggesting stronger vulnerability in males. Our perinatal exposure design (gestation through lactation) complements a prior perinatal HSD study showing dysbiosis/bile acid derangements and NAFLD in weanlings (19). The stronger male responses we observed are consistent with known sex-dependent salt sensitivity and hepatic fibrogenic pathways. Moreover, according to previous studies, there are different degrees of gender differences in the incidence, prevalence, disease manifestations, and drug treatment of diseases, which may also affect the gene expression levels and gene regulation of tissues and organs (77–79). Veiras et al. (80) reported that male db/db mice exhibited higher levels of inflammation and renal immune cell infiltration than females under an HSD, suggesting gender-specific effects of HSD on mice. Additionally, Ceasrine et al. (35) found that a maternal high-fat diet resulted in endotoxin accumulation in fetal tissues, perinatal inflammation, and gender-specific outcomes in offspring. The effect of HSD on the livers of offspring mice in this study also exhibited gender differences. Previous studies have primarily focused on the effects on the maternal liver (38, 81). Taken together, these findings support the concept that maternal diet exerts long-term programming effects on offspring liver health.

These sex-specific hepatic effects are likely mediated by differences in hepatic transcriptional networks and endocrine signaling. Sexually dimorphic expression of transcription factors such as STAT5b, HNF4α, and FOXA1/2 is known to influence lipid metabolism, inflammatory signaling, and fibrotic remodeling (82, 83). Moreover, estrogens exert hepatoprotective effects by modulating pathways such as TEAD1 and attenuating stellate cell activation, while androgens may exacerbate fibrogenic responses (33). These findings align with our observation that male offspring exhibit more robust activation of fibrosis-related and immune-related genes in response to maternal HSD. Although our study did not directly assess hormone levels or manipulate sex hormone pathways, our RNA-seq data revealed enrichment of hormone-responsive pathways, including steroid hormone receptor activity and nuclear receptor transcription coactivator activity, particularly in males. This suggests that maternal HSD may interact with preexisting sex-biased hepatic transcriptional programs, leading to divergent outcomes in liver function and disease susceptibility between the sexes. Future studies will aim to validate these mechanisms at the protein and epigenetic levels, potentially uncovering key regulators that mediate sex-specific hepatic responses to prenatal dietary exposures. Our findings contribute to the broader understanding of the developmental origins of liver disease and underscore the need to consider sex as a critical biological variable in toxicological and nutritional studies.

Fibrosis can develop in various organs, significantly contributing to morbidity and mortality (84, 85). Research has shown that an HSD accelerates cardiac fibrosis formation and alters its expression profiles in C57BL/6 mice (38, 86). Liver fibrosis is characterized by the abnormal proliferation of connective tissue in response to various pathogenic factors, occurring during the repair and healing processes of liver injuries (87, 88). While the current literature predominantly explores the effects of maternal high-fat diets or toxic substance exposures on liver fibrosis in offspring (89, 90), our study demonstrates that maternal HSD also induces liver fibrosis in both male and female offspring. Interestingly, male offspring exhibit a higher incidence of pulmonary fibrosis compared to their female counterparts.

Many diseases induced by high salt are linked to metabolism, inflammation, and autoimmunity (60, 91, 92). For instance, hypertension caused by high salt is considered a component of metabolic syndrome, and excessive salt intake may contribute to metabolic disorders (93). Furthermore, an HSD shifts immune cells from a balanced state to a proinflammatory state. Research indicates that sodium in salt may lead to metabolic reprogramming. Additionally, even a short-term HSD can impair the metabolic fitness and long-term function of Tregs in humans, which is critical in autoimmunity (13). Moreover, an HSD promotes breast cancer progression and lung metastasis by modulating various immune responses (94). The present study also observed that a high-salt maternal diet altered the expression of metabolic and immune-related genes in the livers of offspring mice, alongside increased macrophage infiltration in both male and female offspring. Apoptosis is a natural physiological process, which studies suggest can be triggered by an HSD through activation of pro-apoptotic and pro-inflammatory pathways in the retina (95). Evidence of apoptosis during early embryonic development, particularly in the chicken embryo heart, has been documented (96). Furthermore, apoptosis occurs during liver injury (56, 97). Therefore, this study identified that a maternal HSD altered the expression of apoptosis-related genes in the liver tissue of offspring. Immunofluorescence staining results indicated a higher degree of apoptosis in females compared to males.

CMap is an online tool for drug discovery. The working principle of CMap analysis is to obtain a professional drug research platform for corresponding corrective drugs based on the gene expression differences in target diseases. Using this tool, we obtained a total of 20 top-ranked small-molecule drugs as new drugs for the treatment of liver damage. The gene biological information analysis method used in this study obtained the above drugs. Although preliminary pathological results have confirmed the effectiveness of ketoprofen and BRD-K88741031 in treating liver damage in male and female offspring caused by a high-salt diet, recent studies have shown that ketoprofen plays a key role in improving white fat browning and alleviating diet-induced obesity. Although ketoprofen and BRD-K88741031 treatment improved liver structure and fibrosis histologically, this study did not evaluate their effects on reversing HSD-induced transcriptomic alterations or modulating critical signaling pathways (e.g., TGF-β, NF-κB, or PPAR). This is a significant limitation because functional rescue at the molecular level is crucial for validating therapeutic relevance. Future work will involve transcriptomic and proteomic profiling after treatment to evaluate target engagement and pathway reversal. Additionally, comprehensive preclinical studies, such as dose–response curves, liver function tests, and toxicity assays, are planned to establish the pharmacological and safety profiles of these candidates in a sex-specific context.

Collectively, our findings demonstrate that maternal HSD induces structural and molecular alterations in offspring livers, with more pronounced effects in males, involving fibrotic, inflammatory, and apoptotic pathways. These results carry important translational implications. Globally, average salt intake is nearly double the WHO recommendation, and pregnant women are particularly susceptible to excessive consumption due to taste alterations (5, 22). Epidemiological studies have linked high maternal sodium intake to impaired placental hemodynamics and increased offspring cardiovascular risk, and our data extend these associations by identifying the developing liver as another vulnerable target (98, 99). The early fibrotic and apoptotic changes observed in offspring livers parallel processes underlying pediatric and adult NAFLD progression (19). Furthermore, the male-biased responses align with clinical evidence of sex-specific susceptibility to liver fibrosis and metabolic injury (100). Together, these results highlight a critical window of developmental programming with direct translational relevance, underscoring how maternal nutrition can shape long-term liver health and potentially inform preventive and therapeutic strategies in humans.

While our data suggest that maternal HSD adversely affects the livers of offspring mice, this study has several limitations. Firstly, although mice share key features of mammalian liver biology, species differences—such as circadian metabolism and sodium handling—necessitate cautious extrapolation, and longitudinal human cohort studies are essential to validate the observed programming effects (101). Another, litter sizes were not standardized within the first 1–3 post-natal days. Although we minimized bias by post-weaning weight-based exclusion and balanced sampling across litters, future studies will incorporate early litter homogenization to further eliminate nutritional variability. Secondly, the 4% NaCl model reflects chronic moderate salt exposure, not an acute toxic dose, and thus, better simulates high-risk but realistic human intake (102). However, dose–response studies are needed to identify thresholds for developmental programming. Thirdly, although transcriptomic profiling revealed key sex-specific pathways and regulators, our interpretation remains limited without comprehensive functional validation (103). While histological assessments, F4/80 immunohistochemistry, and cleaved caspase-3 immunofluorescence provided evidence of fibrosis, inflammation, and apoptosis, we did not directly quantify protein expression of transcriptome-derived targets (e.g., PPARα, Ager, TGF-β, and p53). Future studies will incorporate Western blotting, ELISA, and functional assays to confirm molecular pathway involvement and strengthen mechanistic inferences. Additionally, our biochemical evaluation of liver injury was limited. No serum markers (e.g., ALT/AST), hepatic triglyceride levels, or oxidative stress indicators were measured (104). These functional metrics should be included in future investigations to complement histopathological and transcriptomic findings. Finally, our evaluation was restricted to the early post-natal stages (4 weeks of age). It remains uncertain whether the observed changes persist or progress into adulthood (105). We are planning longitudinal studies (8, 16, and 24 weeks) to assess liver histology, fibrosis, metabolic function, and systemic phenotypes, which will clarify the developmental and long-term impact of maternal high-salt exposure. Additional integration of proteomics and metabolomics will further enhance mechanistic insights into sex-specific programming effects.

## 5 Conclusion

In conclusion, an HSD during pregnancy and lactation can impact the liver development of offspring, with male offspring showing greater sensitivity to these effects. Maternal HSD induces significant changes in the liver transcriptome of offspring, potentially leading to liver damage through alterations in metabolic, immune, fibrosis, and apoptosis processes. CMap was employed to identify potential therapeutic drugs for mitigating HSD-induced liver damage in offspring. Therefore, our findings contribute to understanding sex-specific gene–diet interactions underlying maternal HSD-induced liver reprogramming, which can inform the development of effective strategies to prevent liver-related diseases.

## Data Availability

The datasets presented in this study can be found in online repositories. The names of the repository/repositories and accession number(s) can be found in the article/Supplementary material.
