# Supplementary material for: Maternal high-salt intake induces sex-specific liver injury in offspring: integrative transcriptomic and therapeutic investigation
Source: Front Nutr. 2025 Sep 16;12:1657934. doi: 10.3389/fnut.2025.1657934 (PMC12479328; doi:10.3389/fnut.2025.1657934)
Supplement: Supplementary file 11 [file Data_Sheet_1.doc]

***Supporting Information***

**Maternal High-Salt Intake Induces Sex-Specific Liver Injury in Offspring: Integrative Transcriptomic and Therapeutic Investigation**

Xiuli Chen^*^, Rui Chen, Wanyu Song, Li Wang, Haiying Wu^*^

Department of Gynecology and Obstetrics, Henan Provincial People’s Hospital, People’s Hospital of Zhengzhou University, School of Clinical Medicine of Henan University, Zhengzhou, China, 450003

***Corresponding author**

Department of Gynecology and Obstetrics,

Henan Provincial People’s Hospital,

People’s Hospital of Zhengzhou University,

Zhengzhou, Henan, 450003, China

Email: [chenxiuli@zzu.edu.cn](mailto:chenxiuli@zzu.edu.cn) (Xiuli Chen); [wuhaiying@zzu.edu.cn](mailto:wuhaiying@zzu.edu.cn) (Haiying Wu)


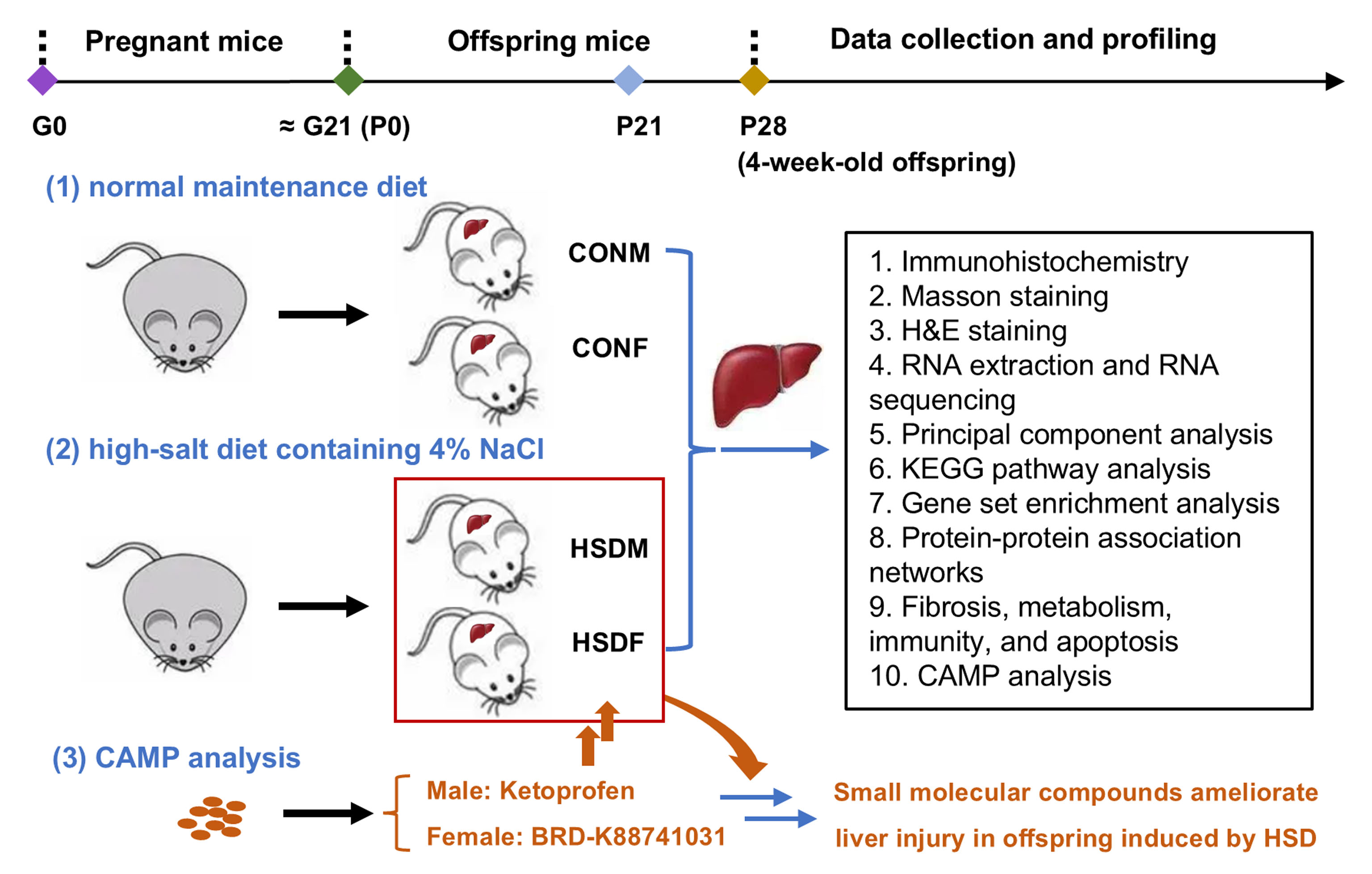


**Figure S1. Schematic overview of the experimental design and study workflow.** Pregnant C57BL/6J mice were fed either a normal maintenance diet (control) or a high-salt diet (HSD) containing 4% NaCl throughout gestation. At postnatal week 4 (P28), male and female offspring were sacrificed, and liver tissues were harvested. Offspring groups included CONM (male control), CONF (female control), HSDM (male HSD), and HSDF (female HSD). Liver samples were subjected to histological analysis (immunohistochemistry, H&E staining, and Masson staining), transcriptomic profiling (RNA extraction and RNA-seq), and bioinformatics analyses (PCA, KEGG, GSEA, and protein-protein interaction networks). CMap analysis was used to identify candidate therapeutic compounds. Sex-specific treatments—ketoprofen for male offspring and BRD-K88741031 for female offspring—were administered based on transcriptomic signatures. The study aimed to assess whether these compounds could mitigate HSD-induced liver injury in a sex-specific manner.


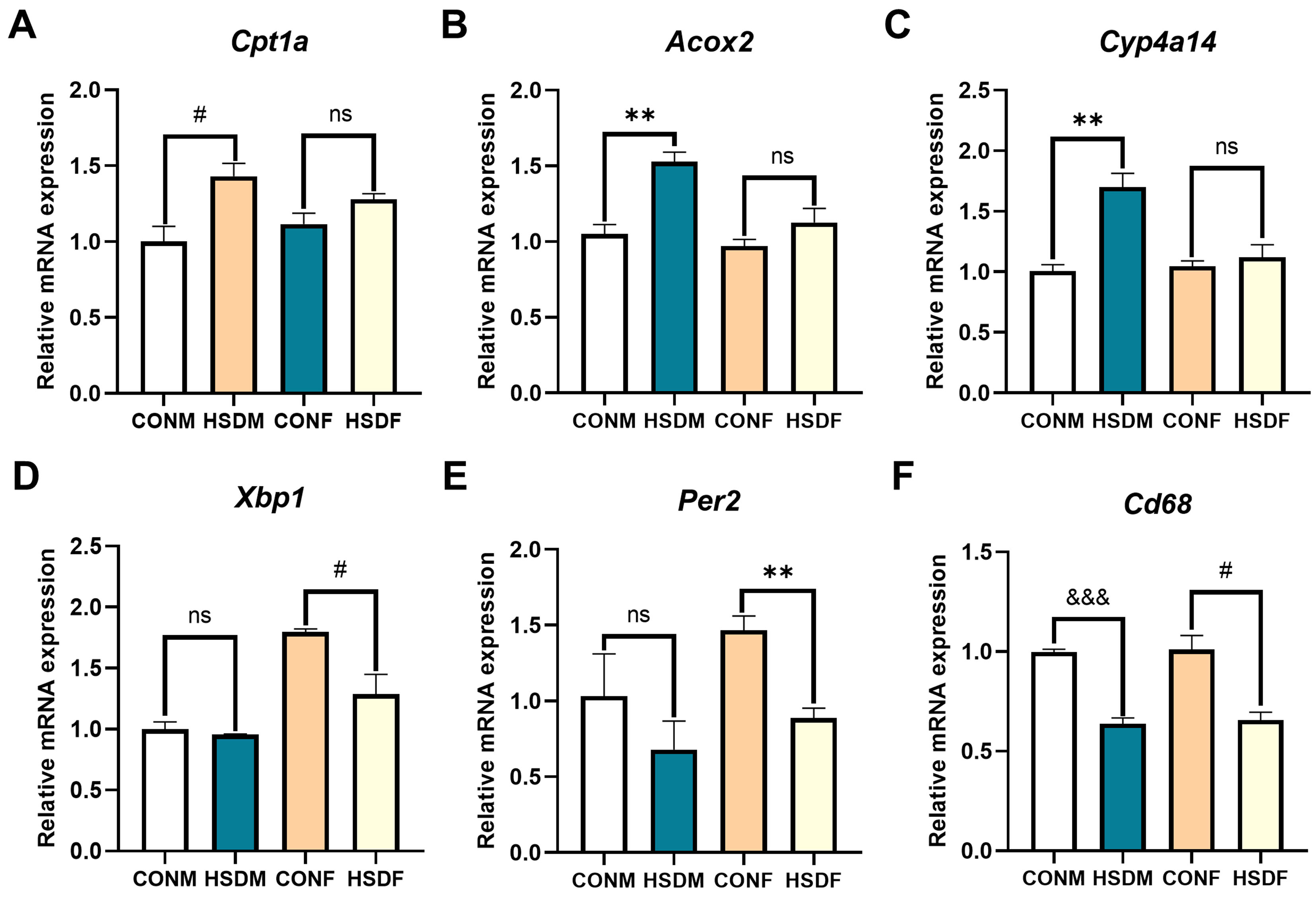


**Figure S2. Effects of maternal HSD treatment on related gene expression in offspring mice, mRNA levels of genes measured by RT-qPCR.** (**A**) *CPT1a* mRNA expression; (**B**) *ACOX2* mRNA expression; (**C**) *CYP4A14* mRNA expression; (**D**) *Xbp1* mRNA expression; (**E**) *Per2* mRNA expression; (**F**) *CD68* mRNA expression. *^#^P* < 0.05, *^**^P* < 0.01, *^&&&^P* < 0.001, ns: no significant difference.


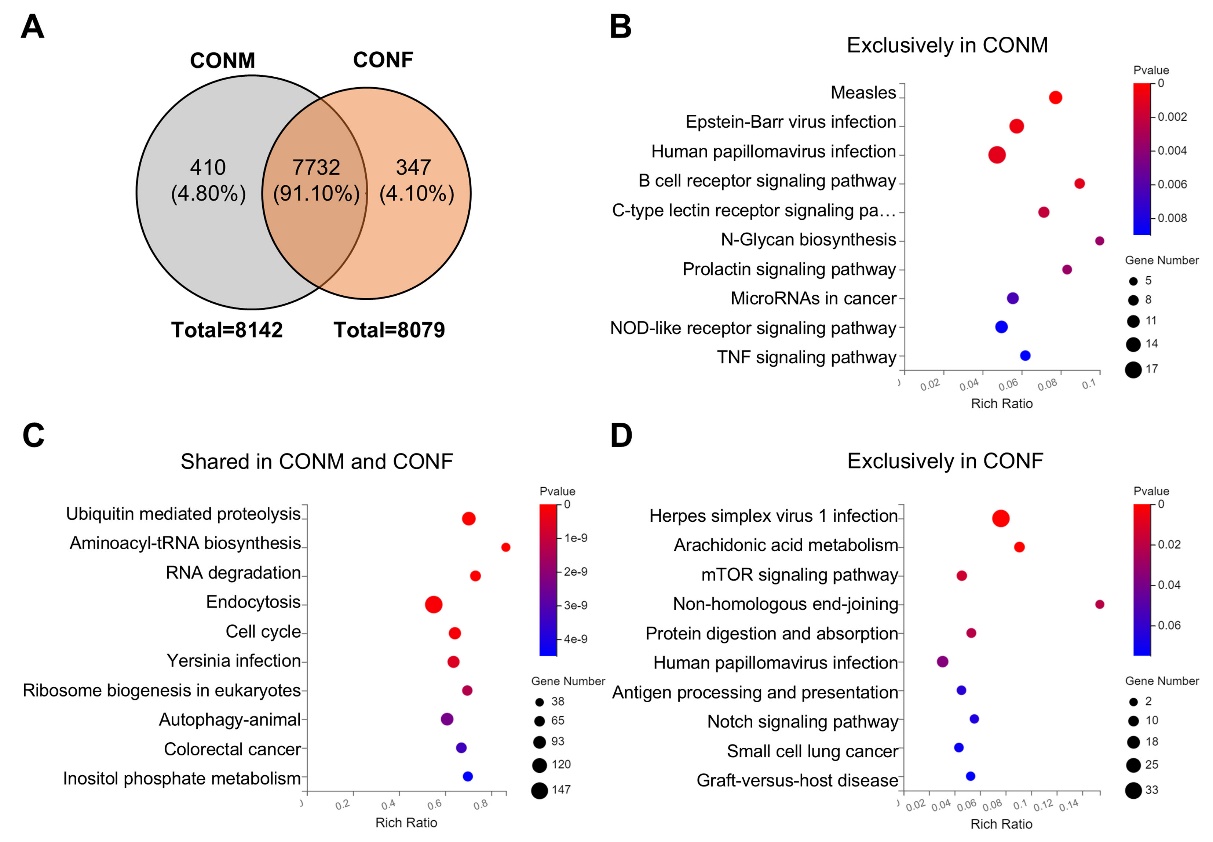


**Figure S3. Maternal HSD alters KEGG pathways with sex-specific effects in the liver of offspring mice.** (**A**) Venn diagram illustrating higher expression genes in the control group and HSD-treated group. (**B-D**) Annotation of top 10 KEGG pathways enriched in higher expression genes between CONM and CONF groups, with *P* < 0.05. *N* = 3 mice per group.


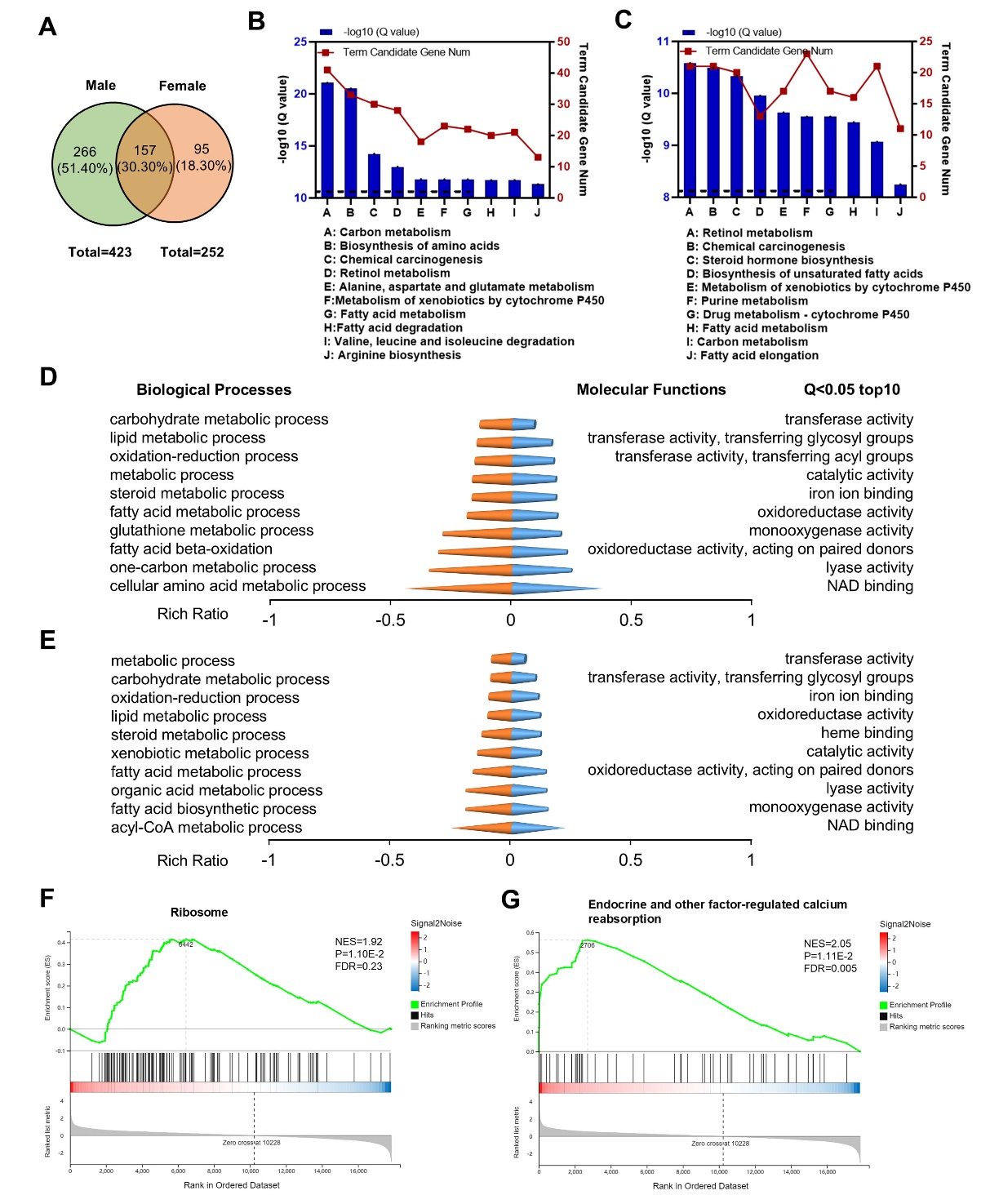


**Figure S4.** **Maternal HSD alters the expression profile of liver metabolism-related genes in offspring mice.** (**A**) Venn diagram depicting liver metabolism-related genes in male and female mice. (**B-C**) KEGG enrichment analysis of DEGs associated with liver metabolism in male and female mouse offspring, with statistical significance at *P* < 0.05. (**D-E**) GO enrichment analysis (molecular functions and biological processes) of DEGs associated with liver metabolism in male and female mouse offspring, with the top 10 displayed in the figures, *Q* < 0.05. (**F-G**) GSEA results show enrichment plots for the KEGG in the liver metabolism associated DEGs of male and female offspring. *N* = 3 mice per group.


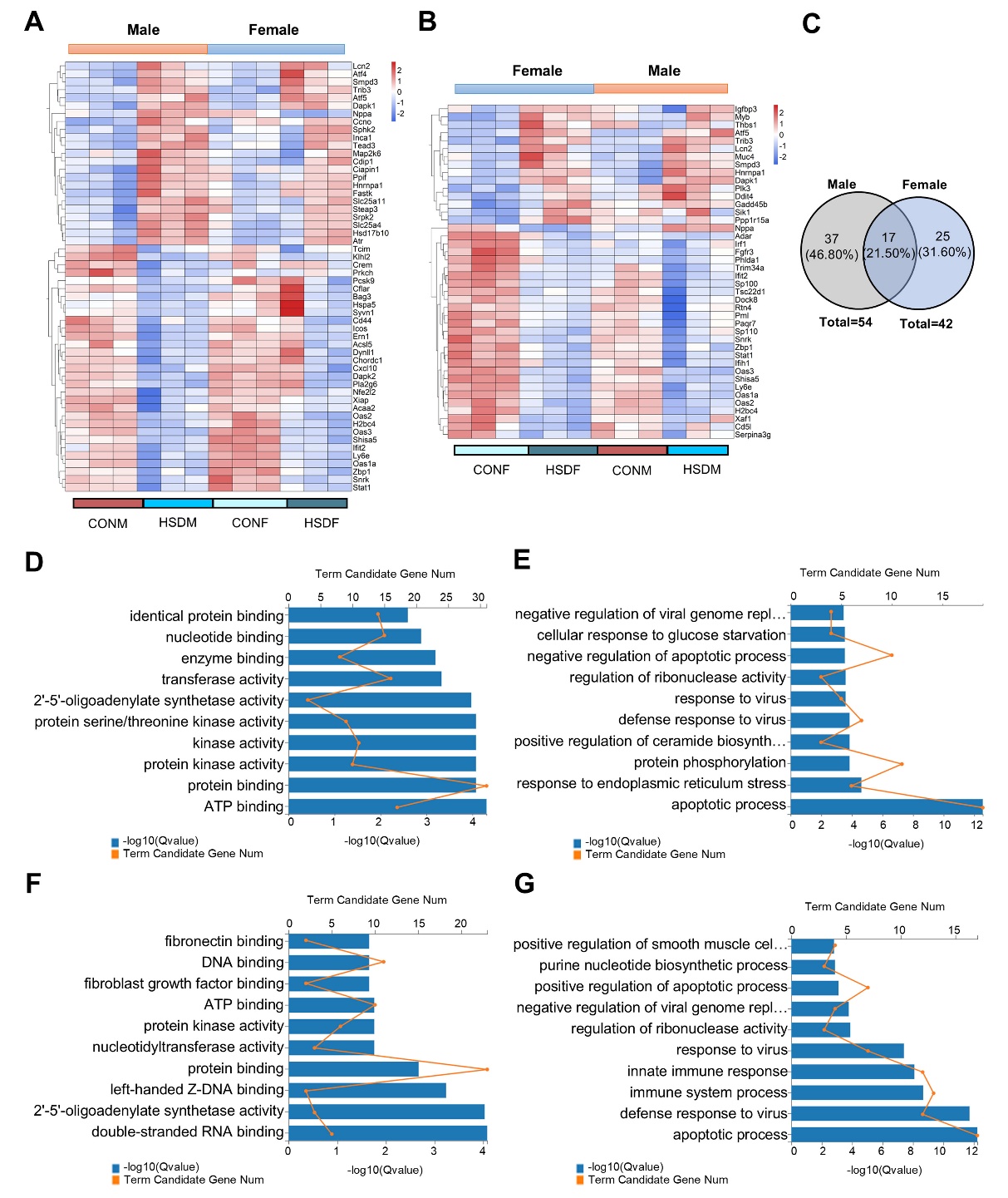


**Figure S5.** **Maternal HSD induces alterations in the expression profile of liver apoptosis-related genes in offspring mice.** (**A-B**) Heatmaps depicting liver apoptosis-related DEGs in male and female mouse offspring. (**C**) Venn diagram showing liver apoptosis-related genes in male and female mice. (**D-G**) GO enrichment analysis (molecular functions and biological processes) of liver apoptosis-related DEGs in male and female mouse offspring. The top 10 pathways are presented, with *Q* < 0.05 significance. *N* = 3 mice per group.
